# Design and Synthesis of New Thiazole Derivatives for Alzheimer’s Disease Therapy as a Potential Cholinesterase Inhibitor; Toxicity Prediction and Molecular Docking Studies and Biological Evaluation

**DOI:** 10.3390/molecules31193568

**Published:** 2026-10-08

**Authors:** Mesut Işık, Gresa Halimi Syla, Abdüllatif Karakaya, Ulviye Acar Çevik, Nour El-Huda Daoud, Muhammet Fırat, Yüksel Öğünç Keçeci, Yusuf Özkay, Zafer Asım Kaplancıklı, Şükrü Beydemir

**Affiliations:** 1 Department of Bioengineering, Faculty of Engineering, Bilecik Şeyh Edebali University, 11230 Bilecik, Turkey; mesut.isik@bilecik.edu.tr; 2 Faculty of Pharmacy, University for Business and Technology, 60000 Prishtina, Kosovo; gresa.halimi@ubt-uni.net; 3 Department of Pharmaceutical Chemistry, Faculty of Pharmacy, Zonguldak Bulent Ecevit University, 67600 Zonguldak, Turkey; a.karakaya@beun.edu.tr; 4 Department of Pharmaceutical Chemistry, Graduate School, Anadolu University, 26470 Eskisehir, Turkey; 5 Department of Pharmaceutical Chemistry, Faculty of Pharmacy, Anadolu University, 26470 Eskişehir, Turkey; uacar@anadolu.edu.tr (U.A.Ç.); yozkay@anadolu.edu.tr (Y.Ö.); zakaplan@anadolu.edu.tr (Z.A.K.); 6 Department of Pharmaceutical Sciences, Faculty of Pharmacy, Al-Ahliyya Amman University, Amman 19328, Jordan; n.daoud@ammanu.edu.jo; 7 Department of Biotechnology, Graduate Education Institute, Bilecik Seyh Edebali University, 11230 Bilecik, Turkey; fenfirat@hotmail.com; 8 Department of Biochemistry, Faculty of Pharmacy, Anadolu University, 26470 Eskisehir, Turkey; yukselogunc@anadolu.edu.tr; 9 The Rectorate of Bilecik Şeyh Edebali University, 11230 Bilecik, Turkey

**Keywords:** thiazole, cholinesterase inhibition, Alzheimer’s disease, molecular docking

## Abstract

In this study, thiazole derivatives **13**–**23** were synthesized as novel cholinesterase inhibitors. Their structures were elucidated using ^1^H-NMR, ^13^C-NMR, and elemental analysis techniques. The binding positions and efficiencies of the designed compounds on the enzyme were investigated through molecular docking studies, and the properties of ADMET were examined using computational methods. Cytotoxicity analyses of the synthesized compounds were performed to screen their inhibitory effects on acetylcholinesterase (AChE) and butyrylcholinesterase (BChE) *in vitro*. It has been determined that these compounds do not exhibit a significant cytotoxic effect on healthy endothelial cells. Although the compounds showed lower efficacy in AChE inhibition compared to the reference compound tacrine (*IC*_50_: 0.136 μM; *K*_I_: 0.46 ± 0.01 nM), they exhibited moderate inhibitory effects. Among these, compounds **16** and **23** were found to be the most potent AChE inhibitors, with *IC*_50_ values of 36.47 µM and 34.65 µM, and *K*_I_ values of 22.20 ± 1.23 and 21.10 ± 1.58 µM, respectively, while compounds **15** and **20** were found to be the most potent BChE inhibitors, with *IC*_50_ values of 17.76 µM and 16.90 µM, and *K*_I_ values of 14.10 ± 0.58 and 13.40 ± 0.48 µM, respectively, on other cholinesterases. These tested compounds can be considered potential candidates for further research in the treatment of Alzheimer’s disease (AD).

## 1. Introduction

The global burden of neurodegenerative disorders has increased considerably, largely as a consequence of the progressive aging of the population. Among these disorders, Alzheimer’s disease (AD) is the predominant cause of dementia and represents a progressive neurological condition characterized by a gradual deterioration of cognitive abilities [1,2]. The clinical manifestations of AD commonly include memory deficits and impairments in language, behavior, visuospatial processing, and, particularly in advanced stages, motor functions [3]. Dysfunction of the cholinergic system in the cerebral cortex is also considered an important feature associated with cognitive decline in AD [2]. The increasing life expectancy observed worldwide has contributed substantially to the growing prevalence of AD, making this disorder an increasingly important public health concern and highlighting the need for effective therapeutic strategies [4]. Current epidemiological estimates indicate that approximately 50 million individuals worldwide are living with severe dementia, with AD accounting for approximately 60–70% of these cases [2]. Moreover, the global number of individuals affected by AD is expected to increase markedly in the coming decades, potentially reaching approximately 100 million by 2050 [1]. Because the disease predominantly affects older adults and progressively compromises cognitive and functional independence, AD constitutes a major challenge for healthcare systems and places a considerable physical, psychological, and social burden on both patients and their caregivers [5,6].

Although the etiology of AD is unknown, it is thought that several factors play a significant role in its onset and progression. AD is characterized by several interconnected pathological processes, including extracellular amyloid-β (A) accumulation and plaque formation, oxidative imbalance, impaired cholinergic signaling associated with reduced acetylcholine (ACh) levels, and tau hyperphosphorylation leading to neurofibrillary tangle formation [1,7]. Consequently, several pathological hypotheses have been put forward in connection with the development of AD. Among these hypotheses, the cholinergic hypothesis stands out [8]. According to the cholinergic hypothesis, one of the fundamental causes of AD is a deficiency in cholinergic neurotransmission [1]. This involves the disruption of neurotransmitters in the synaptic cleft, known as acetylcholine. The enzymes that exacerbate this disruption and trigger AD are acetylcholinesterase (AChE) and butyrylcholinesterase (BChE). These enzymes also contribute to the accumulation of amyloid-β (A) on protein structures in the brain [9]. Furthermore, the accumulation of tau, a microtubule-associated protein, may also contribute to cholinergic neuron degeneration in cases of tauopathy [10].

Unfortunately, there is no cure for AD, and the disease ultimately leads to death. Neuroscientists worldwide are actively conducting research and development into potential drugs to treat AD [6]. When evaluating treatments for neurodegenerative diseases, the use of cholinesterase inhibitors, which block the enzymes that catalyze the breakdown of butyrylcholine (BCh) or ACh, is often considered [10]. Researchers have suggested that AD’s resistance to AChE inhibitors is likely due to BChE activity, which interferes with AChE’s ability to perform or sustain its enzymatic function. Consequently, dual inhibition of AChE and BChE would enhance therapeutic efficacy in the treatment of AD. To date, “rivastigmine” is the only approved dual AChE/BChE inhibitor; the others are single-target molecules [8]. Inhibition of both enzymes is the strategy used to increase ACh levels, and the cholinesterase inhibitor drugs used in current treatment have been developed on this basis [4]. Unfortunately, however, the effects of Food and Drug Administration (FDA) approved drugs are limited, and none of them can halt the progression of the disease. In this context, a growing body of evidence suggests that selective AChE and BChE dual enzyme inhibitors may be more effective in the advanced stages of the disease compared to the drugs used in current treatments. Consequently, cholinesterase inhibitors are regarded as one of the primary targets in the design of multifunctional anti-AD compounds, owing to the beneficial role they play in the early and middle stages of the disease [4,11].

AChE is an enzyme related to BChE, and both cholinesterases exhibit a similar structure, featuring five binding sites within a 20 Å deep binding pocket. This pocket is primarily bounded by the catalytic aromatic site (CAS), which is located near the base, and the peripheral aromatic site (PAS), situated at the entrance to the pocket. The PAS is the first binding site for the substrate; in the CAS, the charged substrate interacts with the aromatic residues. Whilst the hydrolysis reaction takes place in the esteratic region, the oxyanion hole ensures the stabilization of the substrate’s transition state. The final section, the acyl pocket, ensures substrate selectivity. Although AChE and BChE share approximately 65% amino acid sequence identity, structural differences within their active-site regions arise largely from the substitution of aromatic residues by aliphatic amino acids in BChE. These differences are most pronounced in the PAS. PAS (AChE: Tyr341, Asp74, Tyr124, Tyr72, Trp286; BChE: Tyr332, Asp70, Gln119, Ala277, Asn68) and the CAS regions (AChE: Phe338, Tyr337, Tyr133, Trp86; BChE: Phe329, Trp82, Ala328, Tyr128) result in an increase in throat volume of 200 Å, allowing larger ligands to bind to BChE. Although there are fewer aromatic residues in the PAS region of BChE, the Asp70 residue facilitates the transfer of the substrate to the CAS [3,5].

Thiazole-based compounds have attracted considerable interest in medicinal chemistry owing to the broad spectrum of biological activities associated with this heterocyclic scaffold, making it an important structural framework in drug discovery [12]. The thiazole moiety is also a key component of many active pharmaceutical ingredients. Heterocyclic rings are frequently used as the main structural unit in the design of biological compounds. Various studies have demonstrated that compounds containing a thiazole ring are effective against neurodegenerative disorders caused by low levels of cholinergic neurotransmitters [13,14,15,16]. In a study on 1,3-thiazole derivatives by Modrić et al. in 2022, compound **7** was observed to inhibit AChE with an *IC*_50_ value of 91 µM [17]. In BChE inhibition, compound **6** stood out with a value of 195 µM [17]. In a study by Hafez et al. (2023), novel benzothiazole derivatives were synthesized and their AChE inhibition was measured [18]. Compound **1e** showed the highest efficacy with an *IC*_50_ value of 2.92 ± 0.37 µM. Donepezil was used as a positive control (*IC*_50_ value 36.45 ± 8.31 nM) [18]. In another study by Mirjalili et al. in 2024, hydroxycoumarin-based 4-(benzo[d]thiazole-2-yl)phenols were designed and synthesized [19]. AChE inhibition profiles were investigated and Compound **3i** showed the highest activity against AChE with an *IC_5_*_0_ value of 2.7 µM [19]. In another study revealing the multiple target potential of the thiazole core, Hussain et al. synthesized novel thiazolidinedione–thiazole hybrids in 2025. Among these, compound **49** was the most active compound, showing *IC*_50_ values of 0.072 ± 0.002 µM for AChE, 9.81 ± 0.07 µM for BChE and 0.024 ± 0.001 µM for MAO-B. Donepezil (0.055 ± 0.003 µM) was used as the reference drug for AChE inhibition, while safinamide (8.50 ± 0.19 µM for MAO-A, 0.034 ± 0.002 µM for MAO-B) was used as the reference drug for MAO-A and MAO-B [20]. In a study conducted in 2026, Doğan et al. synthesized novel thiazole derivatives and investigated their double cholinesterase inhibition and antioxidant properties. Among these, compound **3** (*IC*_50_ 1.440 ± 0.017 µM for AChE and *IC*_50_ 3.620 ± 0.017 µM for BChE) was found to be the most active compound. In this study, donepezil HCl was used as a positive control (*IC*_50_ values for AChE and BChE were 33.490 ± 0.031 µM and 36.710 ± 0.020 µM, respectively). Compound **3** (80.145 ± 0.021 µM) showed a value close to the reference drug BHT (65.410 ± 0.021 µM) in terms of antioxidant properties according to the DPPH method [21]. These examples shown in the current literature highlight that the thiazole core has an important place in obtaining new target agents that can be the subject of Alzheimer’s treatment and also have antioxidant properties.

This study reports a novel approach to the design and synthesis of new thiazole derivatives **13**–**23** as cholinesterase inhibitors that may demonstrate efficacy as therapeutic agents in the treatment of AD. In this study, 11 new thiazole compounds were synthesized through the design of these new thiazole derivatives. Subsequently, the inhibitory activities of the synthesized compounds against AChE and BChE were determined. Furthermore, to provide a molecular-level perspective on the study, *in silico* studies were conducted, including the prediction of physicochemical properties and molecular docking analyses to investigate the interactions of these compounds with protein domains. Consequently, these compounds demonstrated significant inhibitory activity against both AChE and BChE, acting as anti-Alzheimer’s agents by inhibiting these enzymes. The present study will contribute to future research aimed at developing dual-enzyme inhibitor drugs for the treatment of neurodegenerative diseases caused by AD, utilizing thiazole ring compounds.

## 2. Results and Discussion

### 2.1. Chemistry

The synthesis of the targeted thiazole derivative compounds (**13**–**23**) was carried out in two steps as shown in Scheme 1. In the first step of the synthesis, thiosemicarbazide was refluxed with 1-(3,4-dichlorophenyl)-ethane-1-one as a ketone derivative for 4 h. 2-(1-(3,4-dichlorophenyl)ethylidene)hydrazine-1-carbothiamide (Compound **1**), which will be used in the other step, was successfully obtained. The resulting end product was precipitated in an ice bath and filtered. In the second step, the target compounds (**13**–**23**) were obtained by reacting compound **1** with appropriate acetophenone derivatives (**2**–**12**) by refluxing in ethanol for 4–8 h. The progress of all synthesis steps and the depletion of starting materials were monitored by TLC. The resulting products were precipitated in an ice bath, filtered, and then crystallized from ethanol. The structures of the compounds were elucidated by ^1^H NMR, ^13^C NMR, and elemental analysis (Figures S1–S25). The COSY, HSQC, and HMBC spectra of compound **18** are shown in Figures S13–S15. The COSY spectrum showed the expected correlations between the vicinal aromatic protons of the 4-methoxyphenyl group and between the adjacent protons of the dichlorophenyl ring, supporting the substitution patterns of both aromatic moieties. In the HSQC spectrum, the methoxy protons at δ_H 3.79 correlated directly with the carbon signal at δ_C 55.59, whereas the methyl protons at δ_H 2.32 correlated with the carbon signal at δ_C 14.20. The proton of the thiazole ring showed a direct HSQC correlation with the carbon resonance at δ_C 102.74. The remaining HSQC cross-peaks in the aromatic region connected the proton signals between δ_H 6.97 and 7.95 with their corresponding protonated aromatic carbon atoms. In the HMBC spectrum, the methoxy protons exhibited a diagnostic long-range correlation with the oxygen-substituted aromatic carbon at δ_C 159.27, confirming the 4-methoxyphenyl moiety. In addition, long-range correlations involving the thiazole proton, the hydrazone NH proton, and the methyl group supported the connectivity of the thiazole, hydrazone, and dichlorophenyl fragments. Collectively, the COSY, HSQC, and HMBC results were consistent with and further supported the proposed structure of compound **18**.

### 2.2. Cytotoxicity Assay

The *in vitro* cytotoxic effects of the synthesized thiazole (**13**–**23**) compounds on the human umbilical vein endothelial cell (HUVEC) line, representing a healthy endothelial model, were evaluated utilizing the MTT assay. Cells were exposed to four distinct concentrations ranging from 25 µM to 100 µM, and relative cell viability percentages were calculated in comparison to the control group. Overall findings revealed that none of the tested compounds reduced HUVEC viability below 70%, even at the highest applied concentration of 100 µM (Figure 1). This indicates that the investigated compounds do not exert a marked cytotoxic effect on healthy endothelial cells and demonstrate a reliable safety profile at the cellular level.

### 2.3. Cholinesterase Inhibition Activity

This study investigated the inhibitory properties of novel thiazole derivatives (**13**–**23**) against cholinesterase enzymes. The main objectives of this study were to determine the inhibitory activities of the synthesized derivatives against cholinesterase enzymes associated with AD and to provide preliminary structure–activity relationship data for the future optimization of this compound series. Table 1 presents the *IC*_50_ and *K*_I_ values obtained for the thiazole derivatives and reference inhibitors against the tested cholinesterase enzymes.

Thiazole derivatives (**13**–**23**) were evaluated for their inhibitory effects on the AD-associated cholinesterase enzymes, AChE and BChE. The inhibitory activities of these compounds against cholinesterase enzymes were determined as follows: *IC*_50_ values of 34.65–77.08 µM and *K*_I_ values of 21.10 ± 1.58–46.84 ± 2.63 µM for AChE, and *IC*_50_ values of 16.90–69.30 µM and *K*_I_ values of 13.40 ± 0.48–54.80 ± 2.38 µM for BChE. Although all compounds exhibited lower inhibitory activity against both cholinesterase enzymes than the reference inhibitor tacrine, several derivatives showed measurable inhibition and provided preliminary structure–activity relationship data for further optimization. Tacrine exhibited *IC*_50_ values of 0.136 and 0.207 µM and *K*_I_ values of 0.46 ± 0.01 and 0.33 ± 0.00 µM against AChE and BChE, respectively. These findings indicate that the investigated compounds possess appreciable inhibitory activity within the micromolar concentration range. Furthermore, the determined *K*_I_ values provide valuable insights into the strength of inhibitor–enzyme interactions, thereby contributing to the assessment of inhibitor affinity and selectivity toward the target enzyme. As shown in Table 1, all synthesized derivatives exhibited measurable AChE inhibitory activity in the micromolar range. According to these results, compounds **16** and **23** were found to be the strongest AChE inhibitors with *IC*_50_ values of 34.65 and 36.47, and *K*_I_ values of 21.10 ± 1.58 and 22.20 ± 1.23 µM, respectively, while compounds **15** and **20** were the strongest BChE inhibitors with *IC*_50_ values of 16.90 and 17.76, and *K*_I_ values of 14.10 ± 0.58 and 13.40 ± 0.48 µM, respectively, on other cholinesterases. In contrast, compounds **13** and **14** have a weaker AChE inhibitory effect compared to the other derivatives, with an *IC*_50_ value of approximately 77 and a *K*_I_ value of 47 µM. Specifically, as can be seen from the selectivity index values presented in Table 1, compound **16** exhibited a higher degree of selectivity for AChE compared to all others. Many studies have been conducted in the literature on the enzyme inhibitory potential of thiazole derivatives. The inhibitory potential of the thiazole derivatives synthesized in this study, especially against AChE and BChE cholinesterase enzymes, once again demonstrates that this structural skeleton is an important pharmacophore based on enzyme inhibition.

In one study, based on the cholinergic hypothesis, novel thiazolylhydrazone derivatives were synthesized and their inhibitory potential against cholinesterase enzymes was investigated. These derivatives showed significant inhibitory activity with *IC*_50_ values in the range of 0.031 ± 0.001–>100 µM for AChE, and with *IC*_50_ values of >100 µM for BChE compared to the reference donepezil (*IC*_50_: 0.021 ± 0.001 µM) [22]. Similarly, in another study, a series of benzothiazole-based fused 1,3,4-triazole/1,3,4-thiadiazole hybrid derivatives exhibited *IC*_50_ values of 2.10 ± 0.20–14.10 ± 0.10 µM for AChE, and *IC*_50_ values of 3.10 ± 0.50–15.30 ± 0.10 µM for BChE [23]. In another study, thiazole-sulfonamide derivatives were synthesized, and the inhibitory effect of these derivatives against AChE and BChE was investigated. The compounds exhibited *IC*_50_ values of 0.10–11.40 µM and 0.20–14.30 µM [24]. In the study conducted by Karakaya et al., 5-hydroxymethylfuran-incorporated thiazole-based furan derivatives showed a significant inhibitory effect with *IC*_50_ values between 21.105 and 86.625 µM for AChE, and 6.932 and 34.652 µM for BChE [25].

### 2.4. Molecular Docking

The molecular docking results of the selected compounds showed favorable binding within the catalytic gorge of both AChE and BChE, consistent with their enzyme inhibitory activities (Table 2 and Figure 2). Overall, the predicted binding affinities were higher toward AChE than BChE, as reflected by the more negative Glide GScores and docking scores. Among the investigated derivatives, compound **23** exhibited the strongest predicted affinity for AChE, with a Glide GScore of −8.729 kcal/mol, followed closely by compound **16** (−8.636 kcal/mol), whereas compound **20** showed the most favorable binding toward BChE (Glide GScore = −6.406 kcal/mol), followed by compound **15** (−6.303 kcal/mol). Although the co-ligands displayed the lowest docking scores in both enzymes, the synthesized compounds demonstrated comparable interaction patterns within the active sites, supporting their potential as effective cholinesterase inhibitors.

Within the AChE binding pocket, compound **23** established an extensive interaction network that included three π–π stacking interactions with Trp86, Trp286, and Tyr337, together with hydrogen bonds involving Tyr337 and Glu202. Such simultaneous engagement of residues belonging to both the catalytic active site (CAS) and the peripheral anionic site (PAS) is considered advantageous because it enhances ligand stabilization throughout the active-site gorge and may contribute to stronger enzyme inhibition. This binding mode is further supported by its favorable MM-GBSA binding free energy (−50.18 kcal/mol), suggesting the formation of a thermodynamically stable enzyme–ligand complex. Similarly, compound **16** exhibited a dual-binding orientation by interacting with Trp86, Trp286, Tyr124, and Asp74, yielding a Glide GScore of −8.636 kcal/mol and an MM-GBSA binding energy of −46.65 kcal/mol. The involvement of both catalytic and peripheral aromatic residues indicates efficient occupation of the enzyme gorge and provides a structural rationale for its potent experimental AChE inhibitory activity.

For BChE, compound **20** displayed the best docking performance among the synthesized derivatives, with a Glide GScore of −6.406 kcal/mol and the most favorable MM-GBSA binding energy (−45.44 kcal/mol). Its binding was predominantly stabilized through π–π stacking interactions with the aromatic residues Trp82 and Tyr332, which are essential components of the hydrophobic binding gorge. These interactions likely promote efficient ligand accommodation through aromatic and hydrophobic complementarity. In comparison, compound **15** showed a similar binding orientation within the BChE active site (Glide GScore = −6.303 kcal/mol), where aromatic interactions were reinforced by a hydrogen bond with Asn68 and two halogen bonds involving Gly115 and Glu197. The additional hydrogen- and halogen-bonding interactions may enhance binding specificity and compensate for its slightly less favorable docking score relative to compound **20**.

Comparison of the docking metrics revealed that the selected compounds fall within or exceed the binding affinity range observed for the remaining synthesized derivatives, confirming their selection for further interaction analysis. In particular, compound **23** exhibited the most favorable AChE binding profile, whereas compound **20** showed the strongest predicted interaction with BChE. Despite small differences in Glide GScores, variations in MM-GBSA binding energies indicate that non-covalent interactions beyond docking pose geometry, including hydrogen bonding, π–π stacking, and halogen bonding, contribute substantially to overall complex stability. Collectively, these findings suggest that aromatic stacking interactions with conserved tryptophan and tyrosine residues represent the principal driving force for ligand recognition, while complementary hydrogen- and halogen-bonding interactions further stabilize the complexes. The docking results therefore provide a structural explanation for the experimentally observed cholinesterase inhibitory activities of compounds **23**, **16**, **20**, and **15**, highlighting their ability to occupy the active-site gorge through favorable hydrophobic and polar interactions.

### 2.5. ADMET

The ADMET properties of the most active compounds (**15**, **16**, **20**, and **23**) were predicted using the QikProp module (Table 3). Overall, the selected derivatives exhibited favorable pharmacokinetic profiles, although some parameters deviated slightly from the recommended ranges. The predicted logP values (4.507–6.981) indicated moderate to high lipophilicity, with compound **23** showing the most balanced profile, while compounds **16** and **20** slightly exceeded the recommended limit. As expected, all compounds displayed relatively low aqueous solubility (logS = −6.411 to −8.635), suggesting that their high lipophilicity contributes to limited dissolution.

Despite their low solubility, all compounds demonstrated excellent membrane permeability, with Caco-2 (683–6331 nm/s) and MDCK (3149–10,000 nm/s) values well above the threshold for highly permeable molecules. Accordingly, all derivatives were predicted to have 100% human oral absorption. Furthermore, the calculated logBB values (−0.582 to 0.893) suggest adequate blood–brain barrier penetration, an important characteristic for cholinesterase inhibitors intended for Alzheimer’s disease.

The selected compounds also showed acceptable human serum albumin binding and moderate numbers of predicted metabolic reactions, indicating suitable distribution and metabolic stability. However, all derivatives exhibited predicted hERG inhibition values below the recommended safety threshold, suggesting a potential cardiotoxicity risk that should be confirmed experimentally. Among the evaluated compounds, **23** displayed the most balanced overall ADMET profile owing to its favorable lipophilicity, relatively improved solubility, excellent permeability, and predicted CNS penetration. Overall, the ADMET analysis supports the drug-like potential of these lead compounds while indicating that improving aqueous solubility and reducing hERG liability should be priorities for future optimization.

### 2.6. Structure–Activity Relationship (SAR)

The SAR analysis revealed that the nature and position of substituents on the phenyl ring markedly influenced the AChE inhibitory activity (Table 1). Among the synthesized derivatives, compound **23** bearing 2,4-difluoro substituents exhibited the highest AChE inhibitory activity (*IC*_50_ = 34.65 μM), followed by the 4-chloro derivative **16** (36.47 μM) and the 4-nitro derivative **15** (38.50 μM). These findings suggest that electron-withdrawing substituents, particularly halogens, enhance AChE inhibition, probably by strengthening hydrophobic and aromatic interactions within the enzyme gorge. In contrast, electron-donating (–CH_3_, –OCH_3_) or strongly polar (–CN) substituents resulted in lower AChE activity.

A different trend was observed for BChE inhibition. The 3,4-dichloro derivative **20** displayed the highest potency (*IC*_50_ = 16.90 μM), closely followed by the 4-nitro derivative **15** (17.76 μM). The enhanced activity of compound **20** indicates that increasing hydrophobicity through multiple chlorine substituents favors binding within the larger and more hydrophobic BChE active-site gorge. In comparison, the mono-chloro analog **16** showed significantly weaker BChE inhibition, highlighting the importance of both the number and position of halogen substituents.

Overall, halogenated derivatives emerged as the most promising compounds in this series. While compound **23** demonstrated preferential inhibition toward AChE, compound **20** exhibited the highest selectivity for BChE (*SI* = 2.417). The docking studies further support these observations by showing that the lead compounds establish favorable π–π stacking and hydrophobic interactions with key aromatic residues in the catalytic gorge. Collectively, these findings indicate that appropriate incorporation of electron-withdrawing halogen substituents represents an effective strategy for improving cholinesterase inhibitory activity in this benzimidazole-based scaffold.

## 3. Materials and Methods

### 3.1. Chemistry

All the chemicals employed in the synthetic procedure were purchased from Sigma-Aldrich Chemicals (Sigma-Aldrich Corp., St. Louis, MO, USA) or Merck Chemicals (Merck KGaA, Darmstadt, Germany). Melting points of the obtained compounds were determined by the MP90 digital melting point apparatus (Mettler Toledo, Columbus, OH, USA) and were uncorrected. ^1^H-NMR and ^13^C-NMR spectra of the synthesized compounds were recorded on a Bruker 400 MHz and 100 MHz digital FT-NMR spectrometer (Bruker Bioscience, Billerica, MA, USA) in DMSO-d_6_, respectively. Splitting patterns were designated as follows: s: singlet; d: doublet; t: triplet; m: multiplet in the NMR spectra. Coupling constants (J) were reported in Hertz. All reactions were monitored by thin-layer chromatography (TLC) using Silica Gel 60 F254 TLC plates (Merck KGaA, Darmstadt, Germany).

Synthesis of 2-(1-(3,4-dichlorophenyl)ethylidene)hydrazine-1-carbothioamide derivative (1):

1-(3,4-dichlorophenyl)-ethane-1-one (1 meq) and thiosemicarbazide (1 meq) were dissolved in ethanol and refluxed for 4 h. The end of the experiment was checked by TLC analysis. After the reaction was complete, the solution was cooled in an ice bath to obtain the compound 2-(1-(3,4-dichlorophenyl)ethylidene)hydrazine-1-carbothioamide. The resulting product was filtered.

Synthesis of target compounds (**13**–**23**):

The resulting 2-(1-(3,4-dichlorophenyl)ethylidene)hydrazine-1-carbothioamide derivative (compound **1**) was mixed with appropriate bromoacetophenone derivatives (**2**–**12**) in ethanol under reflux for 4–8 h to obtain the target compounds. The reaction’s completion was monitored by TLC. The product formed at the end of the reaction was cooled in an ice bath. The precipitate was filtered and crystallized from ethanol

2-(2-(1-(3,4-Dichlorophenyl)ethylidene)hydrazineyl)-4-(p-tolyl)thiazole (**13**): M.p.:149.8 °C. Yield: 81%.

^1^H-NMR (400 MHz, DMSO-d_6_): δ: 2.33 (6H, s, CH_3_), 7.22 (2H, d, *J* = 5.84 Hz, Aromatic CH), 7.27 (1H, br.s, Aromatic CH), 6.98 (1H, d, *J* = 6.92 Hz, Aromatic CH), 7.76–7.78 (2H, m, Aromatic CH), 7.95 (1H, s, Aromatic CH), 11.43 (1H, s, NH).

^13^C-NMR (100 MHz, DMSO-d_6_): δ = 14.22 (CH_3_), 21.27 (CH_3_), 125.95, 126.13, 127.27, 127.70, 128.72, 129.66, 131.08, 131.46, 131.80, 137.29, 139.07, 145.78, 166.49, 171.32.

Anal. calcd. For C_18_H_15_N_3_SCl_2_, C, 57.45; H, 4.02; N, 11.17. Found: C, 57.38; H, 4.01; N, 11.20.

2-(2-(1-(3,4-Dichlorophenyl)ethylidene)hydrazineyl)-4-(4-fluorophenyl)thiazole (**14**): M.p.:176.2 °C. Yield: 77%.

^1^H-NMR (400 MHz, DMSO-d_6_): δ: 2.32 (3H, s, CH_3_), 7.25 (2H, t, *J* = 7.92 Hz, Aromatic CH), 7.34 (1H, s, Aromatic CH), 7.68 (1H, d, *J* = 8.40 Hz, Aromatic CH), 7.75 (1H, d, *J* = 8.04 Hz, Aromatic CH), 7.92–7.95 (3H, m, Aromatic CH), 11.45 (1H, s, NH).

^13^C-NMR (100 MHz, DMSO-d_6_): δ = 14.25 (CH_3_), 104.63, 115.92 (d, *J* = 21.31 Hz), 126.13, 127.71, 127.97 (d, *J* = 7.98 Hz), 131.07, 131.51, 131.80, 139.00, 144.39, 150.11, 162.07 (d, *J* = 242.45 Hz), 168.02, 170.03.

Anal. calcd. For C_17_H_12_N_3_SCl_2_F, C, 53.69; H, 3.18; N, 11.05. Found: C, 53.84; H, 3.19; N, 11.07.

2-(2-(1-(3,4-Dichlorophenyl)ethylidene)hydrazineyl)-4-(4-nitrophenyl)thiazole (**15**): M.p.:162.3 °C. Yield: 79%.

^1^H-NMR (400 MHz, DMSO-d_6_): δ: 2.34 (3H, s, CH_3_), 7.70 (1H, d, *J* = 7.84 Hz, Aromatic CH), 7.78 (2H, s, Aromatic CH), 7.97 (1H, s, Aromatic CH), 8.14 (2H, d, *J* = 7.68 Hz, 1,4-disubstituted benzene CH), 8.30 (2H, d, *J* = 8.00 Hz, 1,4-disubstituted benzene CH), (1H, s, Aromatic CH).

^13^C-NMR (100 MHz, DMSO-d_6_): δ = 14.26 (CH_3_), 105.01, 120.80, 125.99, 126.15, 127.72, 128.03, 129.10, 131.09, 131.49, 131.81, 135.15, 139.05, 169.94, 173.90.

Anal. calcd. For C_17_H_12_N_4_O_2_SCl_2_, C, 50.13; H, 2.97; N, 13.76. Found: C, 50.26; H, 2.98; N, 13.80.

2-(2-(1-(3,4-Dichlorophenyl)ethylidene)hydrazineyl)-4-(4-chlorophenyl)thiazole (**16**): M.p.:152.4 °C. Yield: 80%.

^1^H-NMR (400 MHz, DMSO-d_6_): δ: 2.32 (3H, s, CH_3_), 7.46 (1H, s, Aromatic CH), 7.52 (1H, d, *J* = 8.64 Hz, Aromatic CH), 7.70–7.71 (3H, m, Aromatic CH), 7.76 (1H, d, *J* = 8.44 Hz, Aromatic CH), 7.93–7.96 (2H, m, Aromatic CH), 11.51 (1H, s, NH).

^13^C-NMR (100 MHz, DMSO-d_6_): δ = 14.30 (CH_3_), 106.21, 126.18, 127.76, 127.98, 130.25, 131.12, 131.82, 132.69, 135.70, 138.98, 146.84, 151.92, 166.88, 171.96.

Anal. calcd. For C_17_H_12_N_3_SCl_3_, C, 51.47; H, 3.05; N, 10.59. Found: C, 51.63; H, 3.04; N, 10.57.

2-(2-(1-(3,4-Dichlorophenyl)ethylidene)hydrazineyl)-4-(4-cyanophenyl)thiazole (**17**): M.p.:243.5 °C. Yield: 79%.

^1^H-NMR (400 MHz, DMSO-d_6_): δ: 2.38 (3H, s, CH_3_), 7.74–7.75 (2H, m, Aromatic CH), 7.81 (1H, d, *J* = 8.36 Hz, Aromatic CH), 7.93 (2H, d, *J* = 7.52 Hz, 1,4-disubstituted benzene CH), 8.00 (1H, s, Aromatic CH), 8.11 (2H, d, *J* = 7.44 Hz, 1,4-disubstituted benzene CH), 11.59 (1H, s, NH).

^13^C-NMR (100 MHz, DMSO-d_6_): δ = 14.33 (CH_3_), 108.79, 110.07, 119.44, 126.18, 126.59, 127.77, 131.09, 131.62, 131.82, 133.18, 138.90, 139.29, 144.79, 149.47, 170.25.

Anal. calcd. For C_18_H_12_N_4_SCl_2_, C, 55.82; H, 3.12; N, 14.47. Found: C, 55.67; H, 3.13; N, 14.41.

2-(2-(1-(3,4-Dichlorophenyl)ethylidene)hydrazineyl)-4-(4-methoxyphenyl)thiazole (**18**): M.p.:176.2 °C. Yield: 76%.

^1^H-NMR (400 MHz, DMSO-d_6_): δ: 2.32 (3H, s, CH_3_), 3.79 (3H, s, OCH_3_), 6.97 (2H, d, *J* = 7.08 Hz, 1,4-disubstituted benzene CH), 7.17 (1H, br.s, Aromatic CH), 7.68 (1H, d, *J* = 7.48 Hz, Aromatic CH), 7.75 (1H, d, *J* = 8.32 Hz, Aromatic CH), 7.81 (2H, d, *J* = 7.04 Hz, 1,4-disubstituted benzene CH), 7.95 (1H, s, Aromatic CH), 11.41 (1H, s, NH).

^13^C-NMR (100 MHz, DMSO-d_6_): δ = 14.20 (CH_3_), 55.59, 102.74, 114.45, 126.10, 127.32, 127.68, 127.88, 128.08, 131.05, 131.44, 131.80, 139.07, 144.09, 159.27, 169.83.

Anal. calcd. For C_18_H_15_N_3_SOCl_2_, C, 55.11; H, 3.85; N, 10.71. Found: C, 55.29; H, 3.84; N, 10.68.

2-(2-(1-(3,4-Dichlorophenyl)ethylidene)hydrazineyl)-4-(3-bromophenyl)thiazole (**19**): M.p.:148.7 °C. Yield: 78%.

^1^H-NMR (400 MHz, DMSO-d_6_): δ: 2.33 (3H, s, CH_3_), 7.61 (1H, s, Aromatic CH), 7.69 (1H, d, *J* = 8.96 Hz, Aromatic CH), 7.77 (3H, br.s, Aromatic CH), 7.96 (1H, s, Aromatic CH), 8.09 (2H, d, *J* = 7.20 Hz, Aromatic CH), 11.53 (1H, s, NH).

^13^C-NMR (100 MHz, DMSO-d_6_): δ = 14.27 (CH_3_), 106.52, 122.57, 124.82, 126.17, 127.74, 128.66, 130.58, 131.08, 131.30, 131.57, 131.82, 137.46, 138.95, 144.62, 149.54, 170.01.

Anal. calcd. For C_17_H_12_N_3_SCl_2_Br, C, 46.28; H, 2.74; N, 9.52. Found: C, 46.14; H, 2.75; N, 9.55.

2-(2-(1-(3,4-Dichlorophenyl)ethylidene)hydrazineyl)-4-(3,4-dichlorophenyl)thiazole (**20**): M.p.:180.5 °C. Yield: 82%.

^1^H-NMR (400 MHz, DMSO-d_6_): δ: 2.32 (3H, s, CH_3_), 7.59 (1H, s, Aromatic CH), 7.68–7.70 (2H, m, Aromatic CH), 7.75 (1H, d, *J* = 8.44 Hz, Aromatic CH), 7.86 (1H, d, *J* = 8.36 Hz, Aromatic CH), 7.95 (1H, s, Aromatic CH), 8.12 (1H, s, Aromatic CH), 11.49 (1H, s, NH).

^13^C-NMR (100 MHz, DMSO-d_6_): δ = 14.30 (CH_3_), 107.22, 126.01, 126.19, 127.68, 127.76, 130.16, 131.10, 131.36, 131.61, 131.82, 131.92, 138.91, 144.78, 149.98, 166.06, 170.13.

Anal. calcd. For C_17_H_11_N_3_SCl_4_, C, 47.36; H, 2.57; N, 9.75. Found: C, 47.48; H, 2.58; N, 9.72.

2-(2-(1-(3,4-Dichlorophenyl)ethylidene)hydrazineyl)-4-(2,4-dichlorophenyl)thiazole (**21**): M.p.:191.2 °C. Yield: 82%.

^1^H-NMR (400 MHz, DMSO-d_6_): δ: 2.29 (3H, s, CH_3_), 7.31–7.32 (1H, m, Aromatic CH), 7.50 (1H, d, *J* = 8.60 Hz, Aromatic CH), 7.64–7.69 (2H, m, Aromatic CH), 7.72–7.74 (1H, m, Aromatic CH), 7.93 (1H, s, Aromatic CH), 7.96–7.98 (1H, m, Aromatic CH).

^13^C-NMR (100 MHz, DMSO-d_6_): δ = 14.33 (CH_3_), 105.84, 127.97, 128.46, 130.16, 130.26, 131.76, 131.93, 132.82, 132.92, 133.02, 133.48, 136.48, 140.91, 150.99, 165.53, 173.31.

Anal. calcd. For C_17_H_11_N_3_SCl_4_, C, 47.36; H, 2.57; N, 9.75. Found: C, 47.51; H, 2.58; N, 9.71.

2-(2-(1-(3,4-Dichlorophenyl)ethylidene)hydrazineyl)-4-(4-trifluorophenyl)thiazole (**22**): M.p.:163.2 °C. Yield: 76%.

^1^H-NMR (400 MHz, DMSO-d_6_): δ: 2.32 (3H, s, CH_3_), 7.38 (1H, t, *J* = 7.76 Hz, Aromatic CH), 7.49–7.52 (2H, m, Aromatic CH), 7.68 (1H, d, *J* = 8.36 Hz, Aromatic CH), 7.75 (1H, d, *J* = 7.96 Hz, Aromatic CH), 7.88 (1H, d, *J* = 7.20 Hz, Aromatic CH), 7.95 (1H, s, Aromatic CH), 8.09 (1H, s, Aromatic CH), 11.46 (1H, s, NH).

^13^C-NMR (100 MHz, DMSO-d_6_): δ = 14.29 (CH_3_), 107.68, 125.32, 126.07, 126.19, 126.53, 127.11, 127.76, 128.19, 131.10, 131.59, 131.82, 138.95, 153.22, 165.31, 170.23.

Anal. calcd. For C_18_H_12_N_3_SCl_2_F_3_, C, 50.25; H, 2.81; N, 9.77. Found: C, 50.34; H, 2.80; N, 9.74.

2-(2-(1-(3,4-Dichlorophenyl)ethylidene)hydrazineyl)-4-(3,4-dihydroxyphenyl)thiazole (**23**): M.p.:224.2 °C. Yield: 74%.

^1^H-NMR (400 MHz, DMSO-d_6_): δ: 2.33 (3H, s, CH_3_), 7.19 (1H, t, *J* = 8.36 Hz, Aromatic CH), 7.25 (1H, s, Aromatic CH), 7.33–7.38 (1H, m, Aromatic CH), 7.69–7.70 (1H, m, Aromatic CH), 7.75 (1H, d, *J* = 7.72 Hz, Aromatic CH), 7.95 (1H, s, Aromatic CH), 8.04–8.10 (1H, m, Aromatic CH), 11.48 (1H, s, NH).

^13^C-NMR (100 MHz, DMSO-d_6_): δ = 14.27 (CH_3_), 105.03, 112.17, 119.64, 126.16, 127.73, 129.61, 130.75, 131.08, 131.56, 131.82, 135.71, 138.96, 144.10, 150.92, 161.19, 169.42.

Anal. calcd. For C_17_H_13_N_3_SO_2_Cl_2_, C, 51.79; H, 3.32; N, 10.66. Found: C, 51.88; H, 3.33; N, 10.67.

### 3.2. Cytotoxicity Assay

Cell Culture

Human umbilical vein endothelial cells (HUVECs) were cultured using Dulbecco’s Modified Eagle Medium (DMEM; Gibco, Paisley, UK), supplemented with 10% fetal bovine serum and 1% penicillin–streptomycin. The cell cultures were maintained in a standard humidified incubator at 37 °C with an atmosphere containing 5% CO_2_. Routine passaging of the cells was performed at a subcultivation ratio of 1:3.

Cytotoxicity Assay

The *in vitro* cell viability was determined utilizing the 3-[4,5-dimethylthiazol-2-yl]-2,5-diphenyltetrazolium bromide (MTT) assay, based on a slightly modified version of the protocol originally established by Mosmann (1983) [26]. Briefly, HUVECs were seeded into 96-well flat-bottom microtiter plates at a seeding density of 1 × 10^4^ cells per well, suspended in 200 μL of complete culture medium. The plates were incubated for 24 h under standard physiological conditions (37 °C, 5% CO_2_, humidified environment) to allow for proper cell adhesion.

Following attachment, the cells were exposed to the synthesized thiazole compounds at four distinct concentrations (25, 50, 75, and 100 µM) and incubated for an additional 24 h. Upon completion of the treatment phase, 20 μL of sterile-filtered MTT solution (5 mg/mL dissolved in PBS) was introduced to each well, and the microplates were incubated in the dark at 37 °C for 3 to 4 h. The medium containing unreacted MTT was then carefully discarded, and 100 μL of dimethyl sulfoxide (DMSO) was added to every well to dissolve the synthesized purple formazan crystals. To ensure homogeneous solubilization, the plates were subjected to gentle agitation on a shaker for 10–15 min at room temperature. The optical density (OD) of the wells was subsequently measured at a wavelength of 540 nm utilizing a microplate reader (BioTek Instruments, Winooski, VT, USA).

### 3.3. Cholinesterase Activity

Cholinesterase (ChE) activity was determined based on a modified protocol originally described by Ellman et al. (1961) [27]. The enzymatic reactions were performed at 37 °C using acetylthiocholine iodide (ATChI) as the substrate for AChE isolated from electric eel and butyrylcholine iodide (BChI) as the substrate for BChE obtained from horse serum. For each assay, the reaction mixture was prepared in a cuvette containing 100 µL of Tris–HCl buffer (1 M, pH 8.0), 100 µL of DTNB solution (0.01 M), and 50 µL of the corresponding substrate solution (ATChI or BChI; 0.050 M). The assay was initiated by the addition of 50 µL of enzyme solution, corresponding to 5.32 × 10^−3^ U. After a 5 min incubation period, the absorbance was measured spectrophotometrically at 412 nm. Each experiment was conducted in triplicate to ensure the reproducibility of the measurements [28,29].

### 3.4. Molecular Docking Study

Molecular docking studies were performed to investigate the binding modes of the synthesized compounds within the active sites of acetylcholinesterase (AChE) and butyrylcholinesterase (BChE). Docking calculations were carried out using the crystal structures of AChE (PDB ID: 7XN1, 2.85 Å) and BChE (PDB ID: 7Q1M, 2.79 Å), retrieved from the RCSB Protein Data Bank. These structures were selected because they contain co-crystallized tacrine, allowing reliable definition of the active-site binding pocket and validation of the docking protocol. All calculations were performed using Maestro v12.8.117 (Schrödinger Suite 2021-2) under physiological conditions (pH 7.4 ± 1.0), following previously validated protocols [30,31,32,33,34,35,36].

The synthesized compounds were constructed in Maestro and prepared using the LigPrep module. Ionization states were generated with Epik at pH 7.4 ± 1.0, while relevant tautomers, stereoisomers, and low-energy conformers were generated prior to docking.

Protein structures were prepared using the Protein Preparation Wizard. Missing hydrogen atoms were added, bond orders were assigned, and hydrogen-bonding networks were optimized. Protein protonation states were generated using Epik and refined with PROPKA at pH 7.4 ± 1.0. The co-crystallized tacrine was retained only for active-site definition and docking validation, whereas all other ligands were removed. Protein structures were then energy-minimized using the OPLS4 force field with a heavy-atom RMSD convergence criterion of 0.3 Å. Crystallographic water molecules were removed from AChE, whereas structurally relevant water molecules were retained in BChE when appropriate.

Docking grids were generated by centering the receptor box on the co-crystallized tacrine within the catalytic gorge. A grid size of 10 Å in each direction from the ligand centroid was used, with default Glide parameters (van der Waals scaling factor = 1.0; partial charge cutoff = 0.25). Molecular docking was performed using the Glide Standard Precision (SP) protocol, and the optimal binding pose for each compound was selected according to the lowest Glide GScore. Binding interactions with residues located in both the catalytic anionic site (CAS) and peripheral anionic site (PAS) were subsequently analyzed to rationalize the observed cholinesterase inhibitory activities and structure–activity relationships.

### 3.5. ADMET

ADMET (absorption, distribution, metabolism, excretion, and toxicity) properties were predicted using the QikProp module [37] implemented in Schrödinger Suite 2021-2. This tool was used to estimate the physicochemical and pharmacokinetic properties of the synthesized compounds.

## 4. Conclusions

In this study, the newly synthesized thiazole derivatives (**13**–**23**) were designed and evaluated for their inhibitory activities against both AChE and BChE. *In vitro* tests of the compounds showed moderate-to-high inhibition against both cholinesterase enzymes (AChE and BChE). In addition to their anti-Alzheimer’s effect, the cytotoxic properties of the compounds were also investigated on HUVEC lines. The cytotoxic test results also showed that the compounds exhibited a safe cytotoxicity profile. Among the compounds, compound **23**, containing the 3,4,-dihydroxy substituents, showed the highest activity in AChE inhibition (*IC*_50_ = 34.65 µM). Compound **16** followed, also showing high activity (*IC*_50_ = 36.47 µM). Compound **20**, containing a 3,4-dichloro substitute, showed the highest BChE inhibition (*IC*_50_ = 16.90 µM), followed by compound **15**, which also showed high activity (*IC*_50_ = 17.76 µM). Tacrine was used as the reference drug in the cholinesterase inhibition test (*IC*_50_ = 0.136 µM for AChE, *IC*_50_ = 0.207 µM for BChE). These results indicate moderate activity compared to findings from a recent literature review. Some studies have shown that thiazole-based anti-Alzheimer’s agents exhibit inhibition at low micromolar levels. For example, in the study by Hussain et al., it was shown that the AChE *IC*_50_ values of benzimidazole-thiazole derivative compounds ranged from 0.052 ± 0.001 to 9.03 ± 0.36 µM and the BChE *IC*_50_ values ranged from 0.11 ± 0.01 to 17.4 ± 1.08 µM. It was determined that the activity increased when the thiazole ring was enhanced with benzimidazole. In the study by Modrić et al. [17], it was shown that the AChE and BChE inhibitions of 1,3-thiazole derivatives ranged from 91 to 765 µM and 195 to 760 µM, respectively. It is understood that the obtained derivatives (**13**–**23**) show higher activity compared to simple 1,3-thiazole derivatives. In the study by Hafez et al. [18], the study proceeded with the benzothiazole form of thiazole. In the results obtained, most of the compounds showed low activity (>10 µM), while compound **1e** (2.92 ± 0.37 µM) showed the highest activity. It was observed that the activity profile remained moderate, depending on the substituent, by diversifying the thiazole core in this way. In their study, Mirjalili et al. synthesized a series of new benzothiazole rings and investigated their activity [19]. According to their results, the compounds showed AChE inhibition in the range of 2.7 ± 0.10 to 35.8 ± 0.30 µM. Similarly, it is understood that the benzothiazole form of the thiazole ring has a broad and moderate activity profile depending on the substituents. In the study by Doğan et al., new thiazole derivatives were synthesized and their dual cholinesterase inhibition and antioxidant properties were investigated. According to the results obtained, AChE inhibition was observed in the range of 1.440 ± 0.017 to 39.160 ± 0.028 µM, and BChE inhibition in the range of 3.620 ± 0.017 to 40.850 ± 0.023 µM [17,18,19,20,21]. A comparison of recent thiazole-focused anti-Alzheimer studies in the literature highlights the thiazole ring as a focal point in studies targeting Alzheimer’s disease. Molecular docking studies supported the results obtained from *in vitro* tests. The strong π-π interactions and complementary halogen interactions of the precursor compounds highlighted their binding ability to the enzyme’s active site. ADMET predictions also emphasized that the compounds exhibited good critical pharmacokinetic properties such as blood–brain barrier penetration and membrane permeability. Taken together, although these thiazole derivatives bearing a dihalogenated phenyl ring exhibited lower cholinesterase inhibitory activity than tacrine, the findings provide preliminary structure–activity relationship data that may guide the further optimization of this chemical scaffold.

## Figures and Tables

**Scheme 1:**
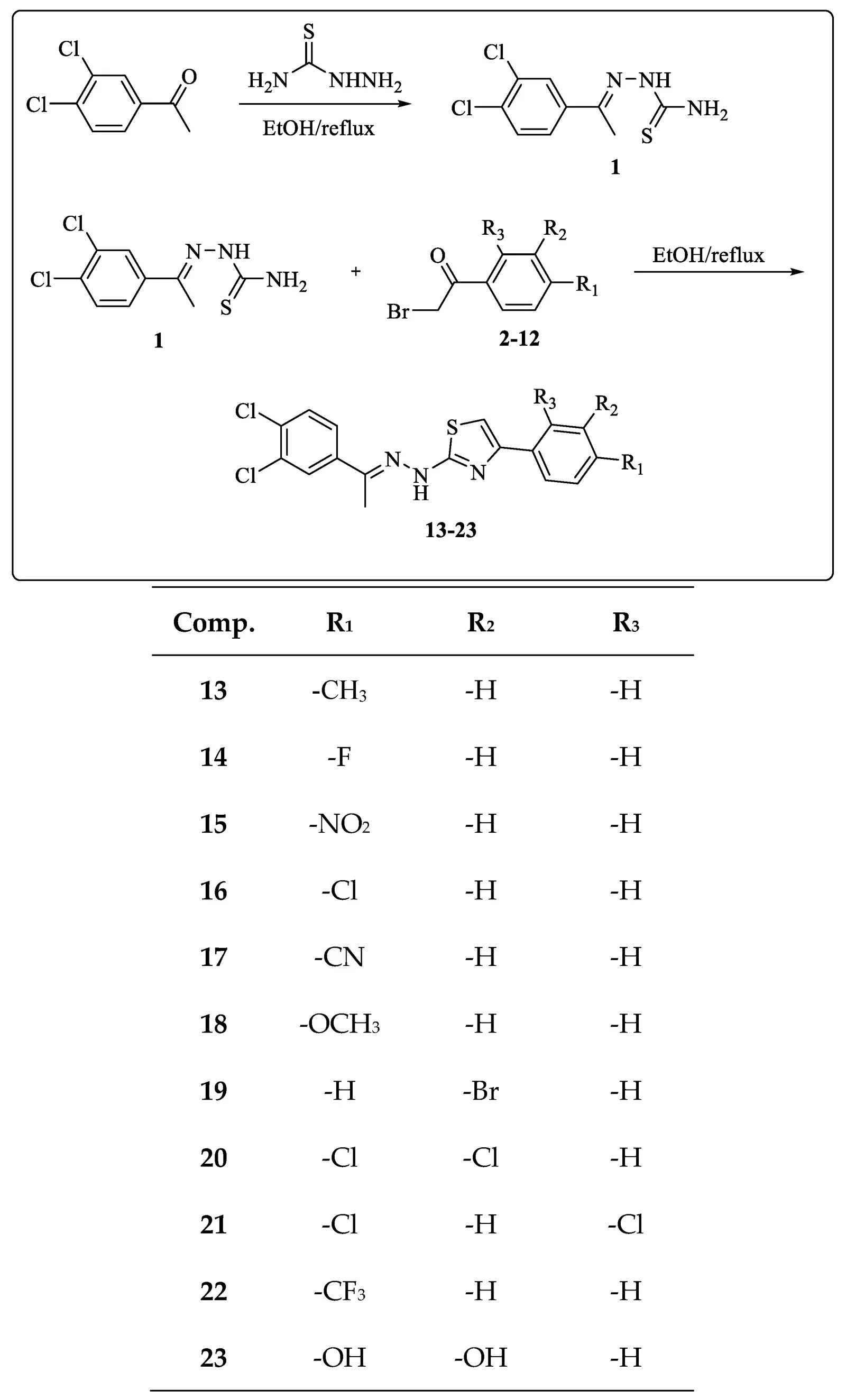
General procedure for synthesis of the final compounds **13**–**23**.

**Figure 1:**
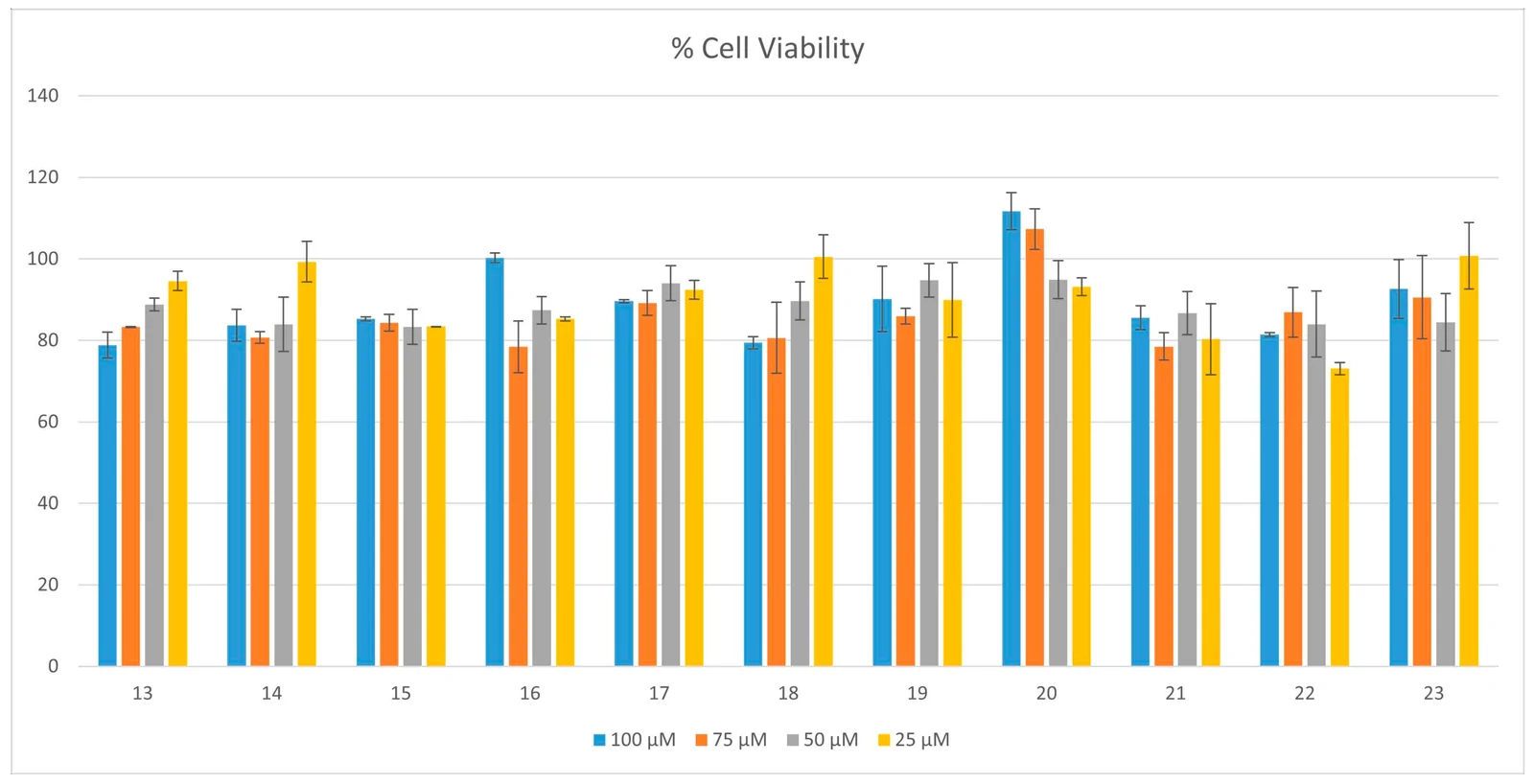
Cell viability of HUVECs for 24 h.

**Figure 2:**
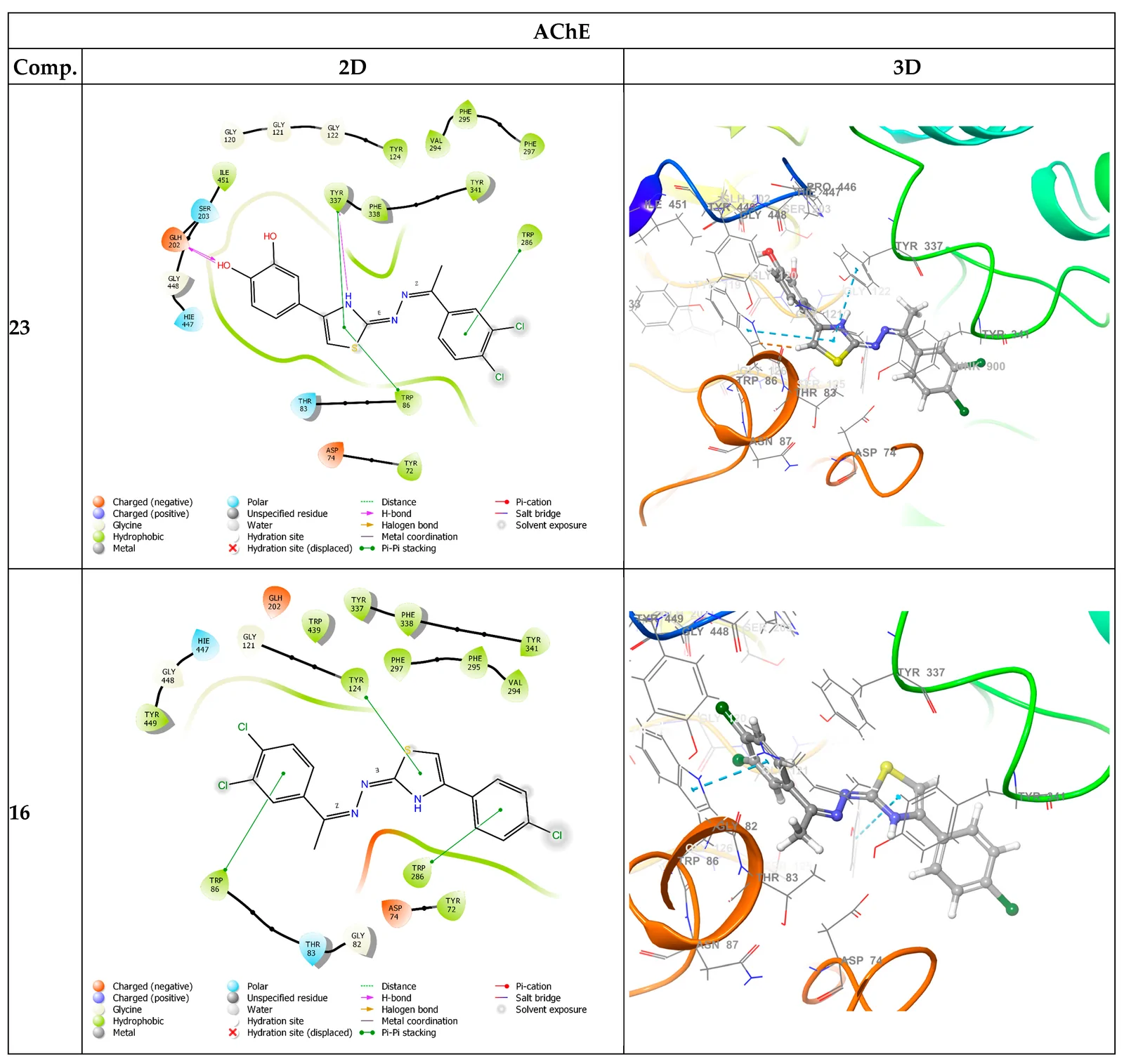

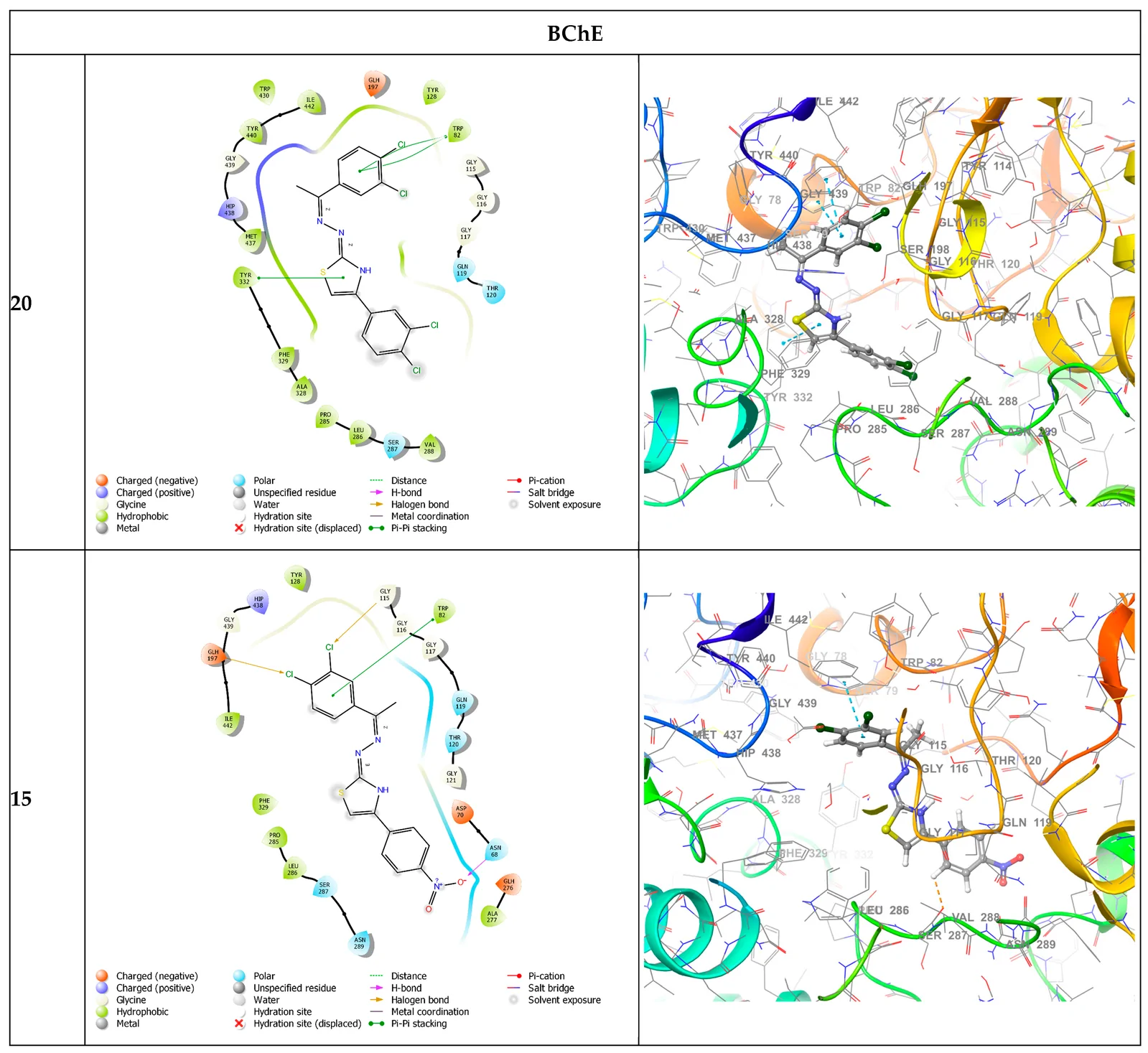
2D and 3D protein–ligand interaction profiles of selected compounds (**15**, **16**, **20** and **23**) within the active sites of AChE (PDB ID: 7XN1) and BChE (PDB ID: 7Q1M). In AChE, compound **23** forms three π–π stacking interactions with Trp86, Trp286 and Tyr337, along with hydrogen bonding with Tyr337 and Glh202 (Glide GScore: −8.729 kcal/mol), indicating simultaneous engagement of the catalytic and peripheral sites. Compound **16** interacts primarily with Trp86, Trp 286 and Tyr 124Asp74 (−8.636 kcal/mol), suggesting a dual-binding mode. In BChE, compound **20** shows strong aromatic stabilization through π–π stacking with Trp82, Tyr332 (−6.38 kcal/mol). Similarly, compound **15** interacts with key aromatic residues within the hydrophobic gorge, with Trp86 additionally forming a hydrogen bond with Asn68, as well as two halogen bonds with Gly115 and Glh197 (−6.123 kcal/mol). Overall, ligand binding is predominantly governed by aromatic and hydrophobic interactions, supporting stable accommodation within the enzyme active-site gorge.

**Table 1:** *IC*_50_ and *K*_I_ values of compounds **13**–**23** against AChE and BChE.

Comp.	AChE ^a^				BChE ^b^				
	*IC*_50_ (μM)	*R* ^2^	*K*_I_ (μM)	*R* ^2^	*IC*_50_ (μM)	*R* ^2^	*K*_I_ (μM)	*R* ^2^	*SI* *
**13**	77.02	0.945	46.81 ± 2.63	0.964	27.72	0.936	21.90 ± 1.32	0.957	2.136
**14**	77.08	0.942	46.84 ± 1.59	0.950	26.65	0.923	21.10 ± 1.16	0.943	2.218
**15**	38.5	0.874	23.40 ± 0.87	0.953	**17.76**	0.929	**14.10 ± 0.58**	0.939	1.659
**16**	**36.47**	0.929	**22.20 ± 1.23**	0.947	69.30	0.911	54.80 ± 2.38	0.929	0.405
**17**	69.30	0.934	42.20 ± 2.08	0.951	30.13	0.954	23.80 ± 1.25	0.972	1.773
**18**	69.30	0.944	42.20 ± 3.05	0.959	31.50	0.920	24.90 ± 1.48	0.951	1.694
**19**	57.75	0.880	35.10 ± 1.87	0.933	31.50	0.921	24.90 ± 1.11	0.945	1.409
**20**	53.30	0.939	32.40 ± 1.65	0.962	**16.90**	0.955	**13.40 ± 0.48**	0.968	2.417
**21**	69.30	0.958	42.20 ± 2.42	0.987	28.87	0.919	22.80 ± 0.68	0.942	1.850
**22**	57.75	0.869	35.10 ± 1.73	0.923	24.75	0.963	19.60 ± 1.28	0.976	1.790
**23**	**34.65**	0.851	**21.10 ± 1.58**	0.971	25.66	0.928	20.30 ± 0.98	0.947	1.039
**Tacrine ^c^**	0.136	0.978	0.46 ± 0.01	0.964	0.207	0.954	0.33 ± 0.00	0.973	1.394

**^a^** AChE, acetylcholinesterase; **^b^** BChE, butyrylcholinesterase; **^c^** positive control. * The selectivity index (*SI*) was determined using the equation *SI* = *K*_I_ (AChE)/*K*_I_ BChE). The most favorable *IC*_50_ and *K*_I_ values are shown in bold.

**Table 2:** Molecular docking results of the most potent compounds for AChE and BChE (**15**, **16**, **20** and **23**). The remaining synthesized compounds are summarized as ranges (kcal/mol).

Compound	AChE				BChE			
	Glide GScore	Glide Energy	Docking Score	MM-GBSA ΔGbind	Glide GScore	Glide Energy	Docking Score	MM-GBSA ΔGbind
15	−8.614	−50.298	−8.435	−40.45	**−6.303**	**−50.705**	**−6.123**	**−39.61**
16	**−8.636**	**−49.461**	**−8.610**	**−46.65**	−6.127	−47.424	−6.101	−37.42
20	−7.548	−46.958	−7.660	−27.15	**−6.406**	**−51.714**	**−6.380**	**−45.44**
23	**−8.729**	**−52.090**	**−8.670**	**−50.18**	−5.916	−47.738	−5.857	−40.09
Co-ligand	−9.116	−37.610	−9.116	−63.09	−7.539	−51.984	−7.535	−68.02
Other compounds	−8.58 to −7.69	−53.25 to −47.78	−8.58 to −7.66	−51.27 to −28.80	−6.27 to −5.51	−51.98 to −44.83	−6.24 to −4.63	−47.40 to −32.83

**Table 3:** Predicted ADMET properties of the synthesized compounds (QikProp analysis). For the most active compounds (**15**, **16**, **20** and **23**).

Description	Recommended Range	15	16	20	23
Octanol/water partition (logPo/w)	−2.0–6.5	5.394	6.576	6.981	4.507
Aqueous solubility (logS)	−6.5–0.5	−7.492	−8.031	−8.635	−6.411
Conformation-independent solubility (CIlogS)	−6.5–0.5	−7.324	−7.507	−8.228	−6.6
HERG K^+^ channel blockage log(IC50)	>−5 (concern below −5)	−6.315	−6.27	−6.188	−6.101
Caco-2 permeability (nm/sec)	<25 poor; >500 great	750.005	6331.284	6084.329	683.407
Brain/blood partition (logBB)	−3.0–1.2	−0.498	0.783	0.893	−0.582
MDCK permeability (nm/sec)	<25 poor; >500 great	3483.234	10,000	10,000	3149.647
Skin permeability (logKp)	−8.0–−1.0	−2.284	−0.547	−0.705	−2.344
Predicted metabolic reactions	1–8	4	3	3	5
Binding to human serum albumin (logKhsa)	−1.5–1.5	0.972	1.13	1.241	0.545
Human Oral Absorption	1 (low), 2 (medium)	1	1	1	1
Human Oral Absorption percentage (%)	>80% high	100	100	100	100
Max. transdermal transport rate	-	0.000068	0.001048	0.000197	0.000692

## Data Availability

The data supporting the findings of this study are provided in the manuscript and the Supplementary Information files.

## Supplementary Materials

The following supporting information can be downloaded at https://www.mdpi.com/article/10.3390/molecules31193568/s1: Figures S1–12, S16–25: ^1^H and ^13^C NMR spectra of the synthesized compounds; Figures S13–S15: COSY, HMBC, and HSQC spectra of compound **18**.

## Abbreviations

The following abbreviations are used in this manuscript:

Aβ	Amyloid-beta
ACh	Acetylcholine
AChE	Acetylcholinesterase
AD	Alzheimer’s Disease
ADMET	Absorption, distribution, metabolism, excretion, and toxicity
ATChI	Acetylthiocholine Iodide
BCh	Butyrylcholine
BChE	Butyrylcholinesterase
BChI	Butyrylcholine Iodide
ChE	Cholinesterase
CAS	Catalytic aromatic site
DMEM	Dulbecco’s Modified Eagle Medium
DMSO	Dimethyl Sulfoxide
FDA	Food and Drug Administration
HUVEC	Human Umbilical Vein Endothelial Cells
J	Coupling constants
MTT	3-[4,5-dimethylthiazol-2-yl]-2,5-diphenyltetrazolium bromide
OD	Optical Density
PAS	Peripheral Aromatic Site
SAR	Structure–Activity Relationship
*SI*	Selectivity index
