# Supplementary material for: Design and Synthesis of New Thiazole Derivatives for Alzheimer’s Disease Therapy as a Potential Cholinesterase Inhibitor; Toxicity Prediction and Molecular Docking Studies and Biological Evaluation
Source: Molecules. 2026 Oct 8;31(19):3568. doi: 10.3390/molecules31193568 (PMC13666930; doi:10.3390/molecules31193568)
Supplement: Supplementary file 1 [file molecules-31-03568-s001.zip › molecules-4515628-supplementary.pdf]

Design, synthesis of New Thiazole Derivatives for Alzheimer Disease Therapy as a Potential Cholinesterase Inhibitör; Toxicity prediction and Molecular Docking studies and Biological Evaluation

Mesut Işık[1], Gresa Halimi Syla[2], Abdüllatif Karakaya[3,4], Ulviye Acar Çevik[5], Nour El-Huda Daoud[6], Muhammet Fırat[7], Yüksel Öğünç Keçeci[8], Yusuf Özkay[5], Zafer Asım Kaplancıklı[5,9], Şükrü Beydemir[8]*

[1] Department of Bioengineering, Faculty of Engineering Bilecik Şeyh Edebali University, 11230, Bilecik, Turkey; mesut.isik@bilecik.edu.tr
[2] Faculty of Pharmacy, University for Business and Technology, Prishtina, Kosovo; gresa.halimi@ubt-uni.net
[3] Department of Pharmaceutical Chemistry, Faculty of Pharmacy, Zonguldak Bulent Ecevit University, 67600 Zonguldak, Turkey; a.karakaya@beun.edu.tr
[4] Department of Pharmaceutical Chemistry, Graduate School, Anadolu University, Eskisehir, Türkiye; a.karakaya@beun.edu.tr
[5] Department of Pharmaceutical Chemistry, Faculty of Pharmacy, Anadolu Universty, 26470 Eskişehir, Turkey; uacar@anadolu.edu.tr, yozkay@anadolu.edu.tr, zakaplan@anadolu.edu.tr
[6] Department of Pharmaceutical Sciences, Faculty of Pharmacy, Al-Ahliyya Amman University, Amman, Jordan; noordaoud@hu.edu.jo
[7] Department of Biotechnology, Graduate Education Institute, Bilecik Seyh Edebali University, 11230, Bilecik, Turkey; fenfirat@hotmail.com
[8] Department of Biochemistry, Faculty of Pharmacy, Anadolu University, 26470 Eskisehir, Turkey; yukselogunc@anadolu.edu.tr, sukrubeydemir@anadolu.edu.tr
[9] The Rectorate of Bilecik Şeyh Edebali University, 11230, Bilecik, Turkey; zakaplan@anadolu.edu.tr

* Correspondence: sukrubeydemir@anadolu.edu.tr

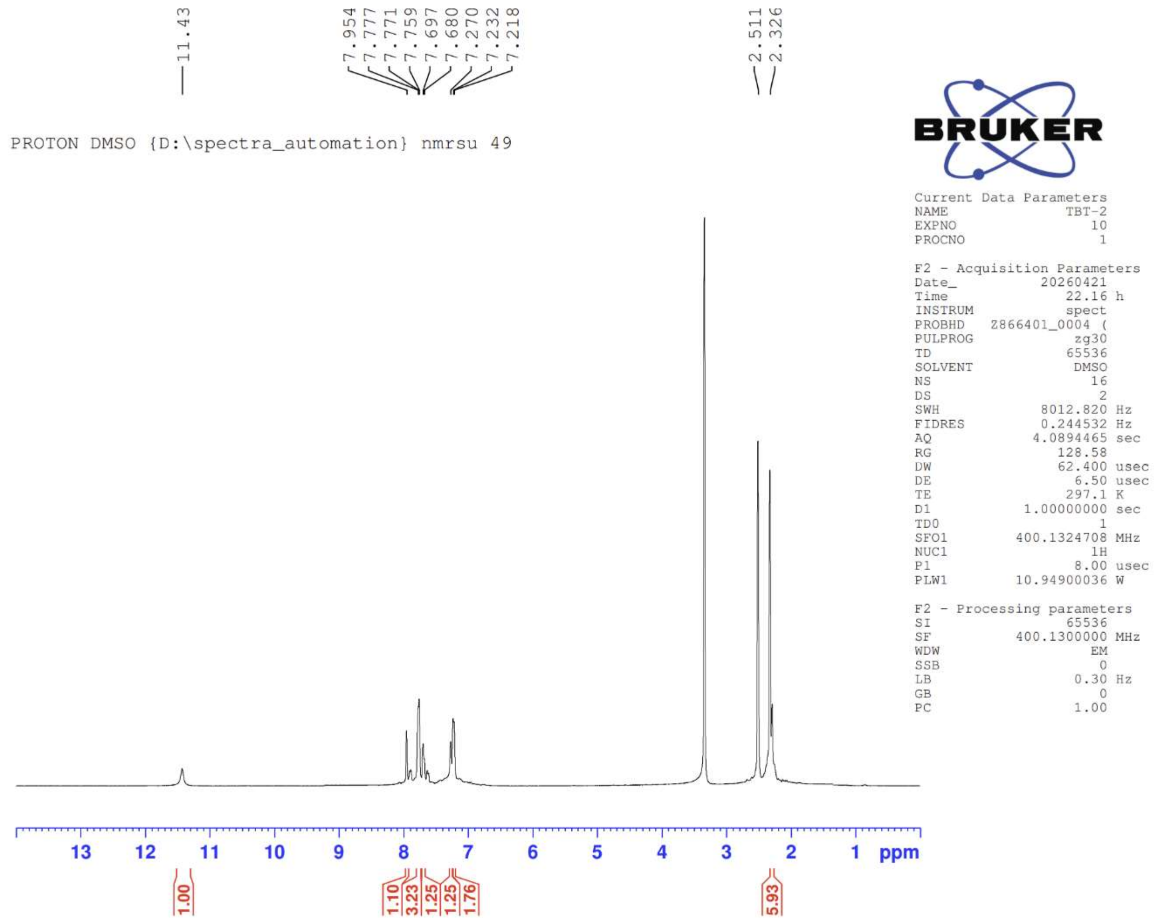

Figure S1. ^{1}H-NMR spectrum of compound **13**

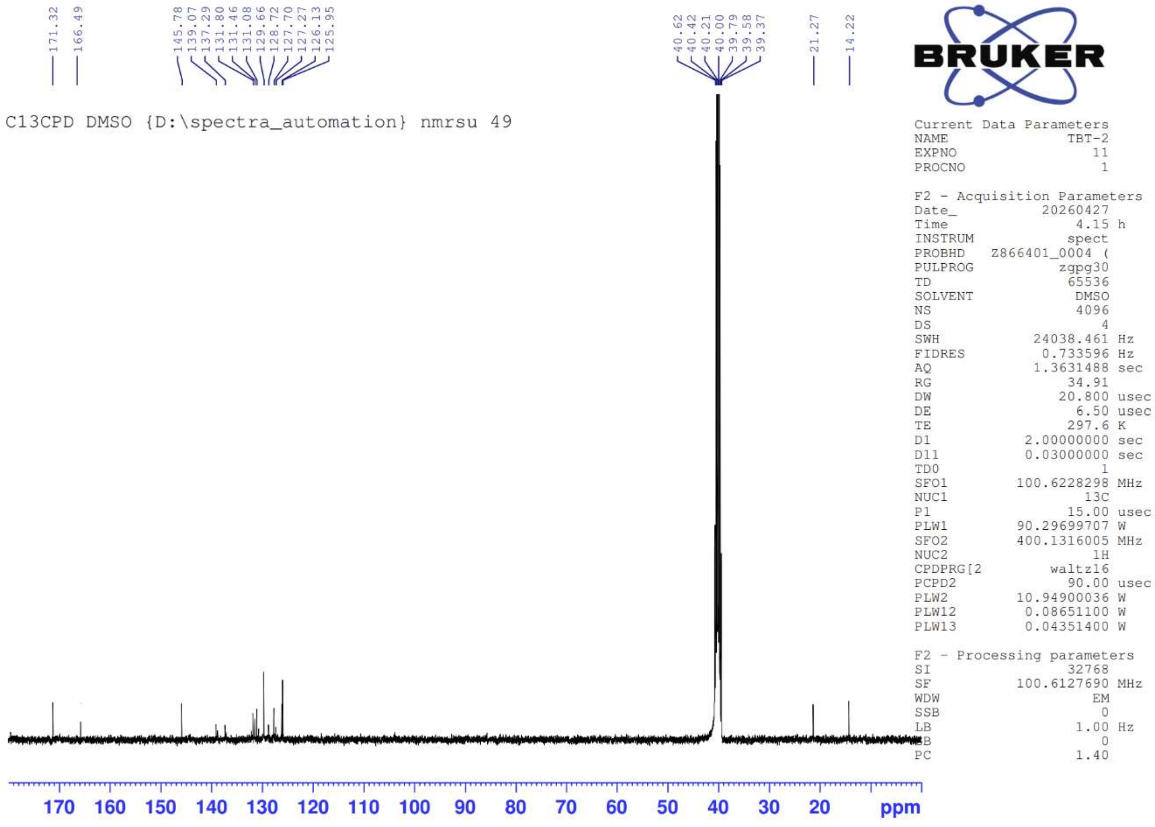

Figure S2. ^{13}C-NMR spectrum of compound **13**

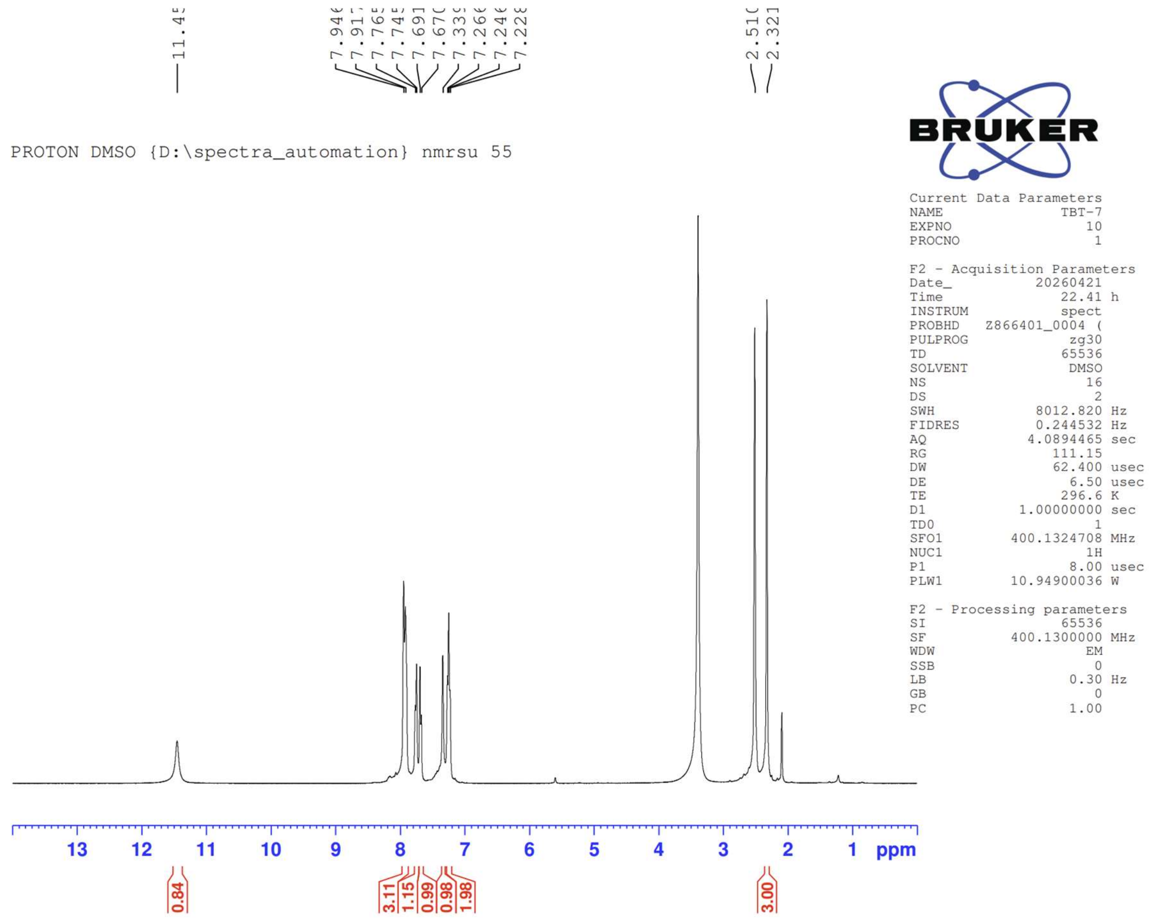

Figure S3. ^{1}H-NMR spectrum of compound **14**

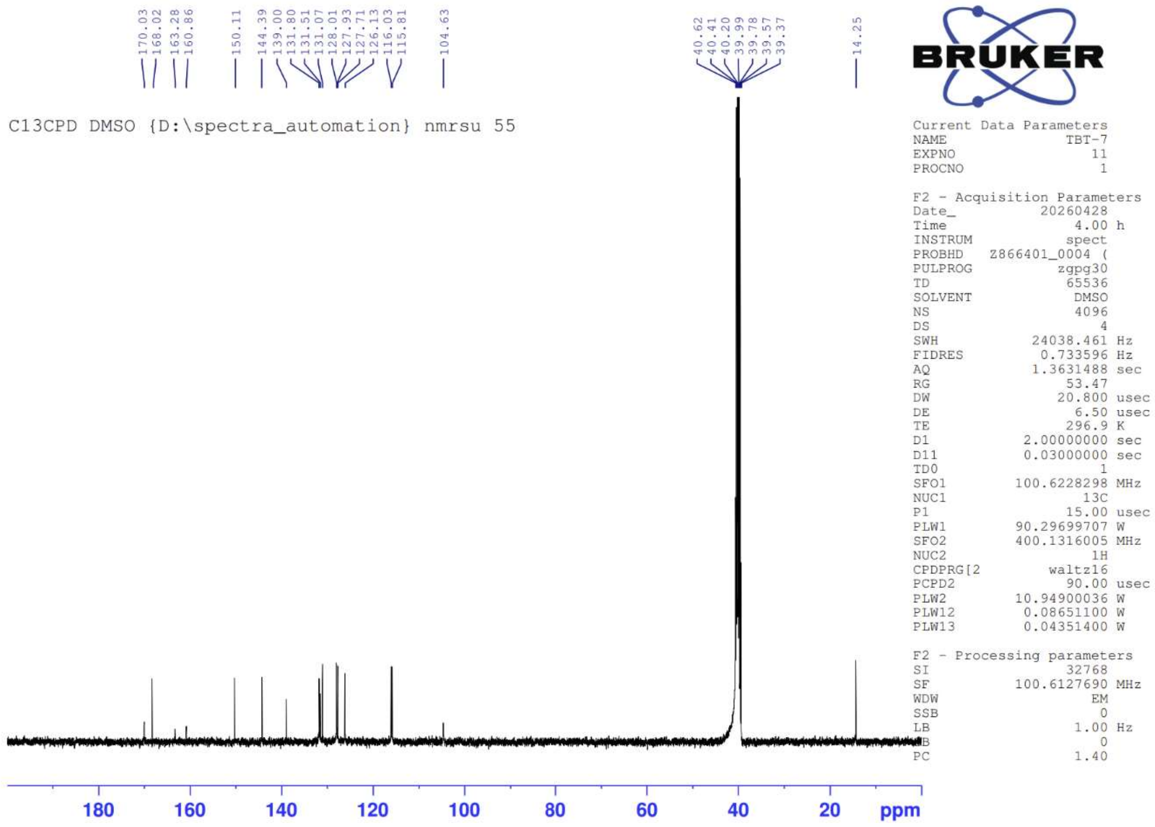

Figure S4. ^{13}C-NMR spectrum of compound **14**

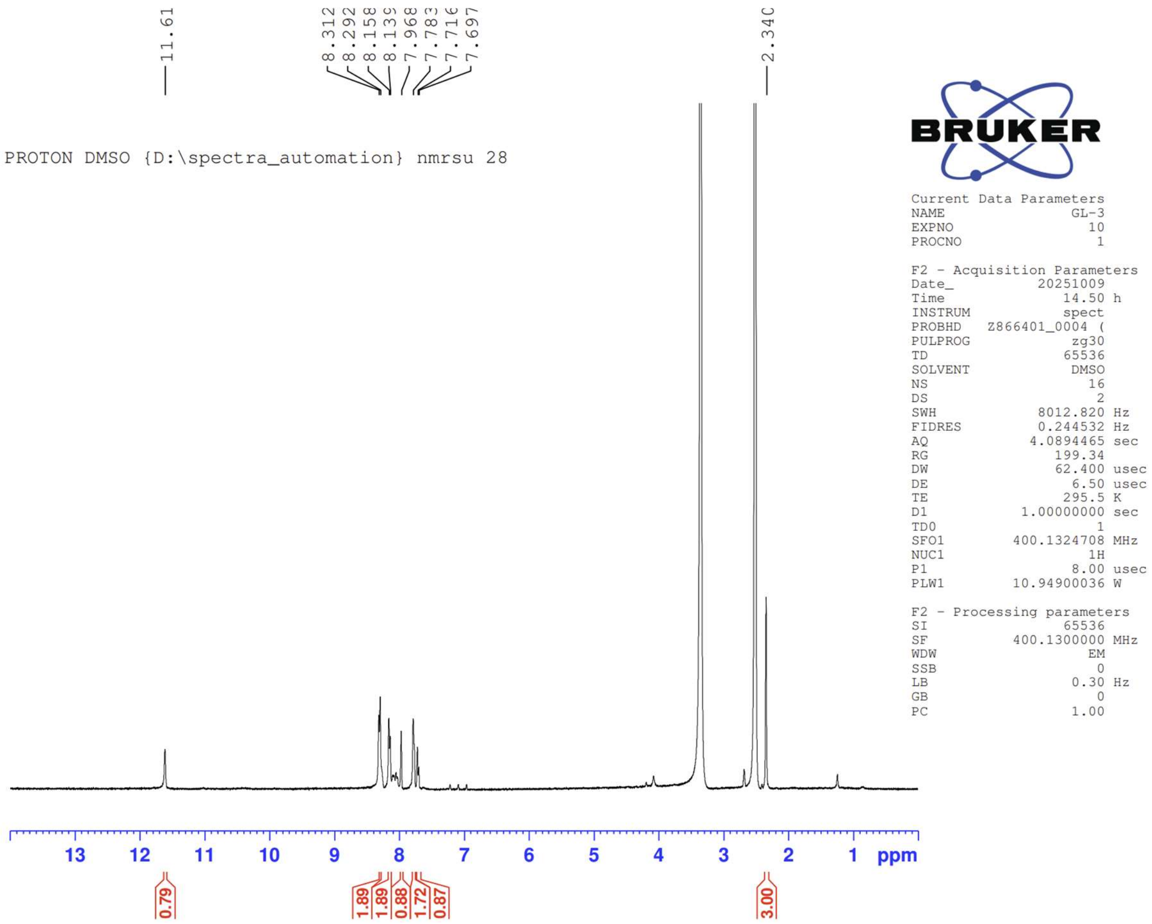

Figure S5. ^{1}H-NMR spectrum of compound **15**

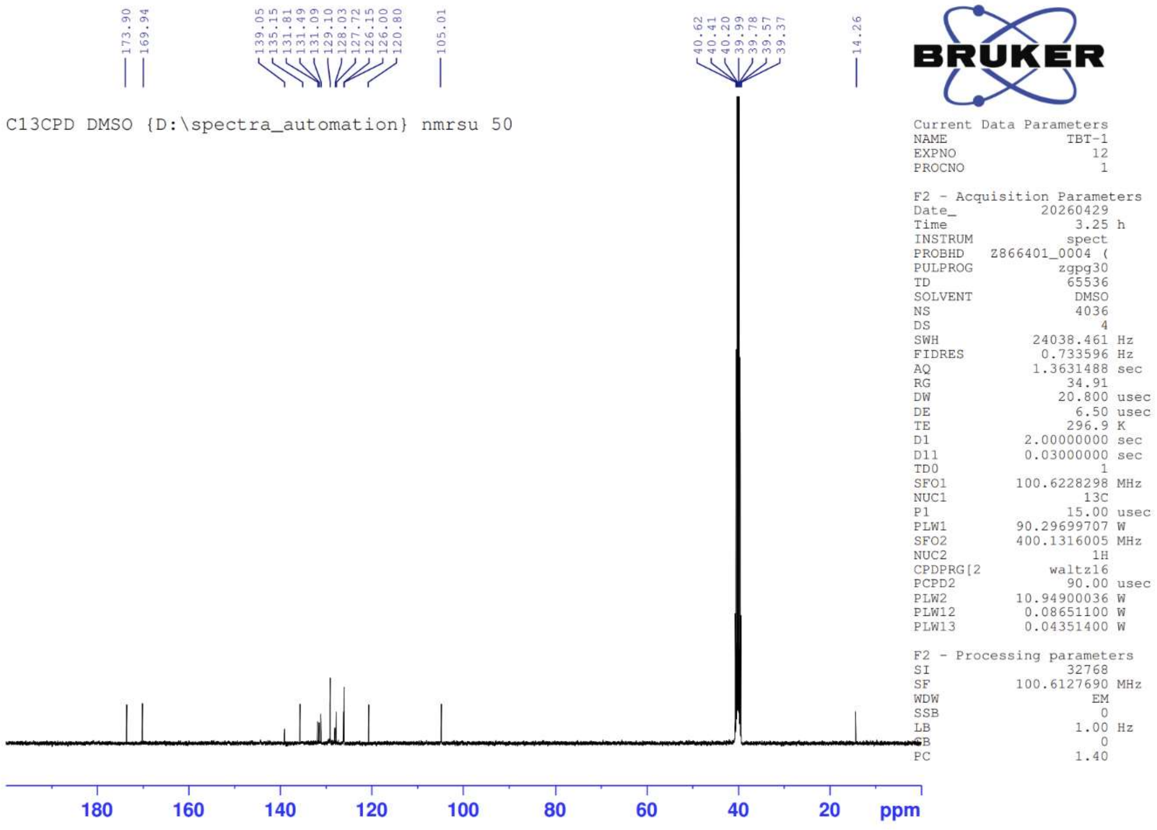

Figure S6. ^{13}C-NMR spectrum of compound **15**

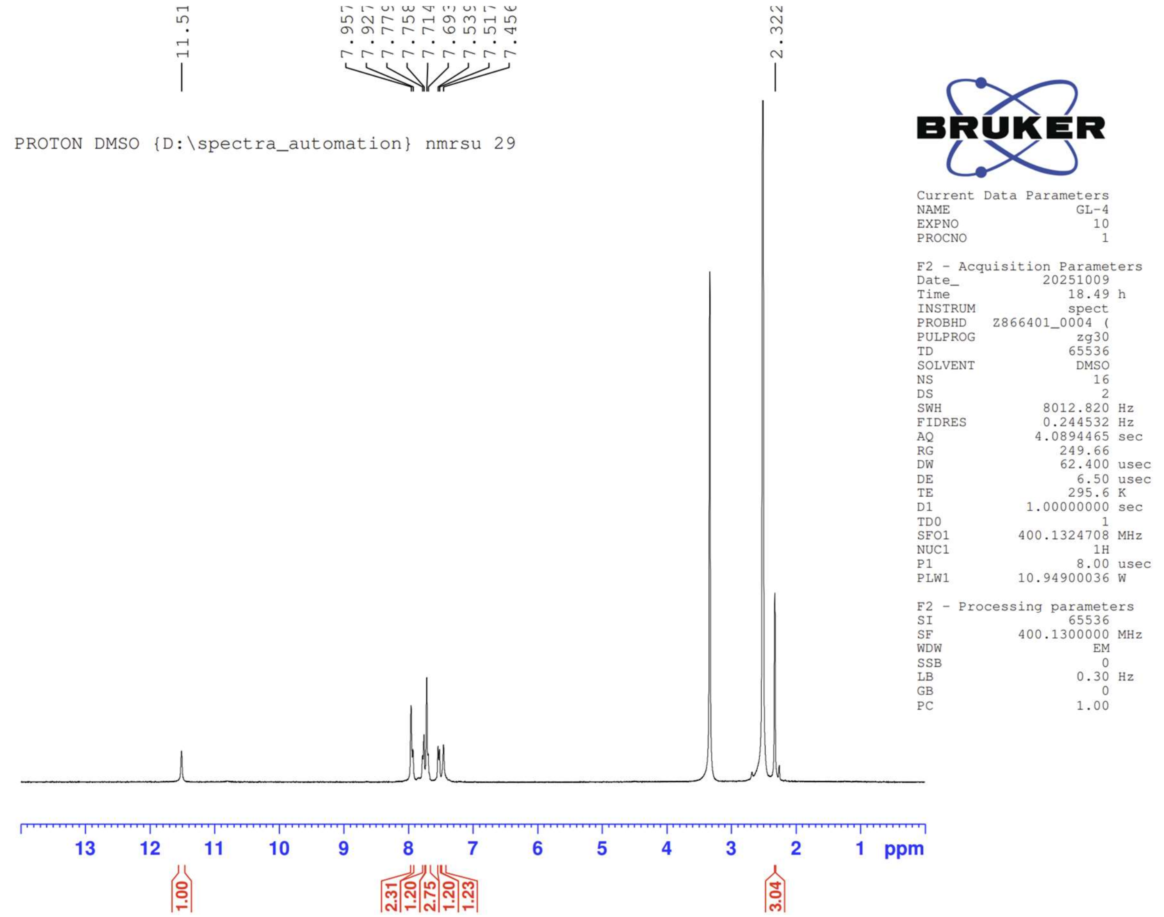

Figure S7. ^{1}H-NMR spectrum of compound **16**

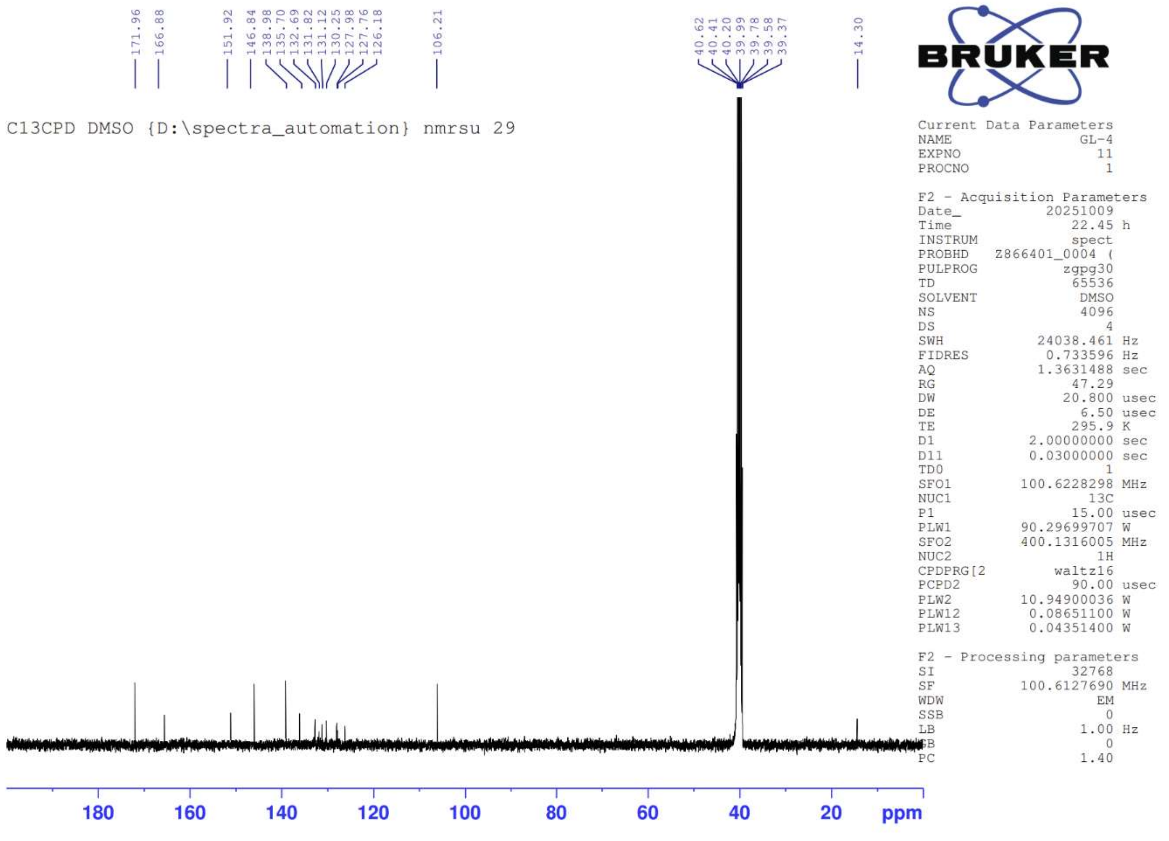

Figure S8. ^{13}C-NMR spectrum of compound **16**

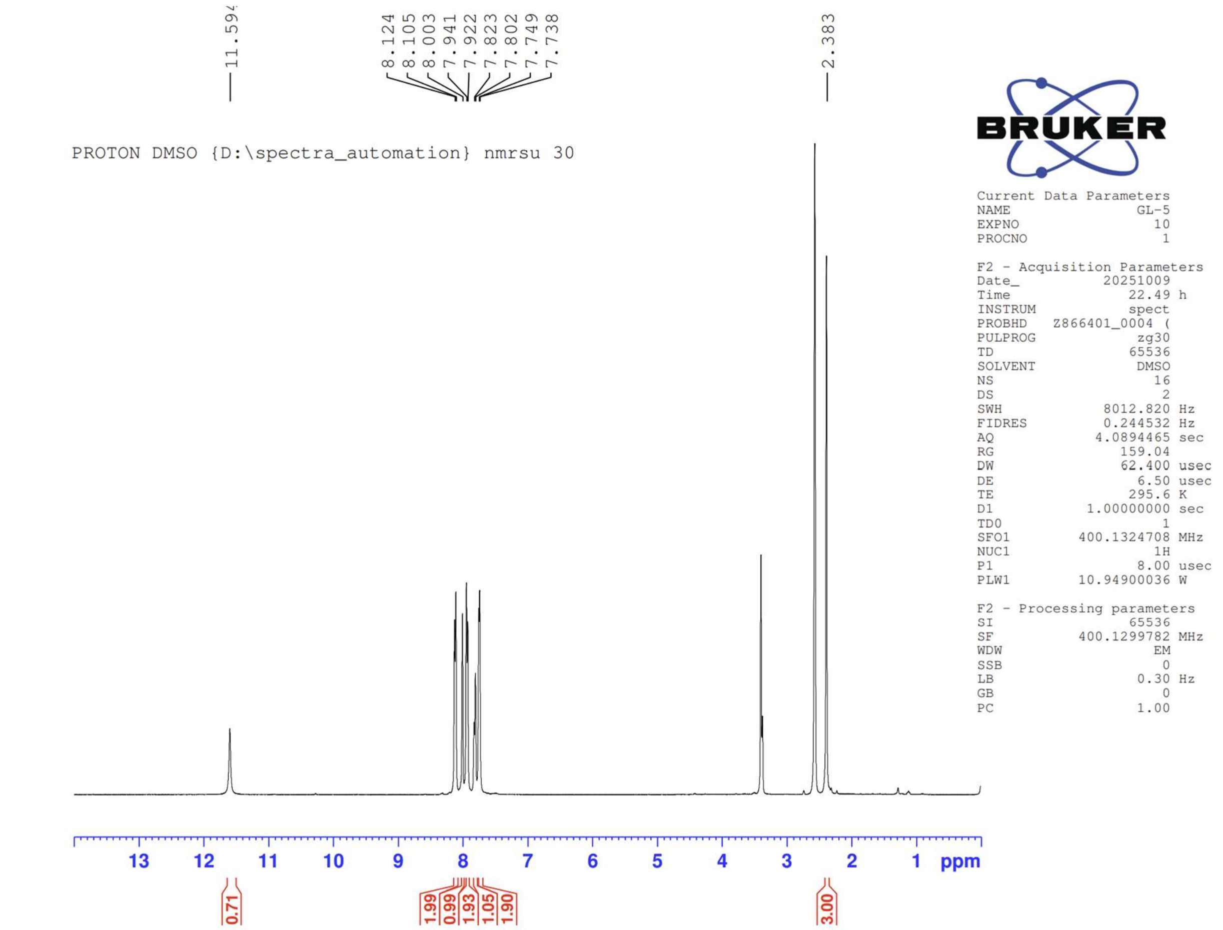

Figure S9. ^{1}H-NMR spectrum of compound **17**

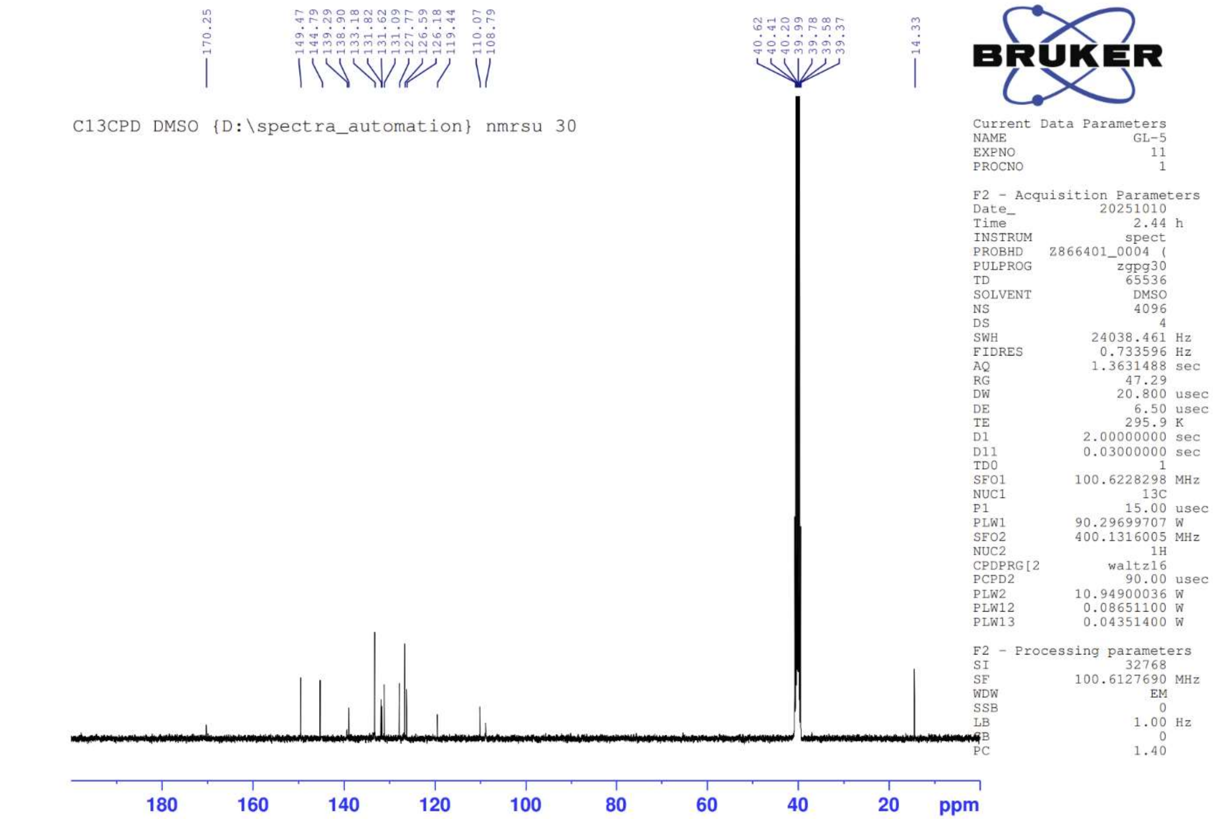

Figure S10. ^{13}C-NMR spectrum of compound **17**

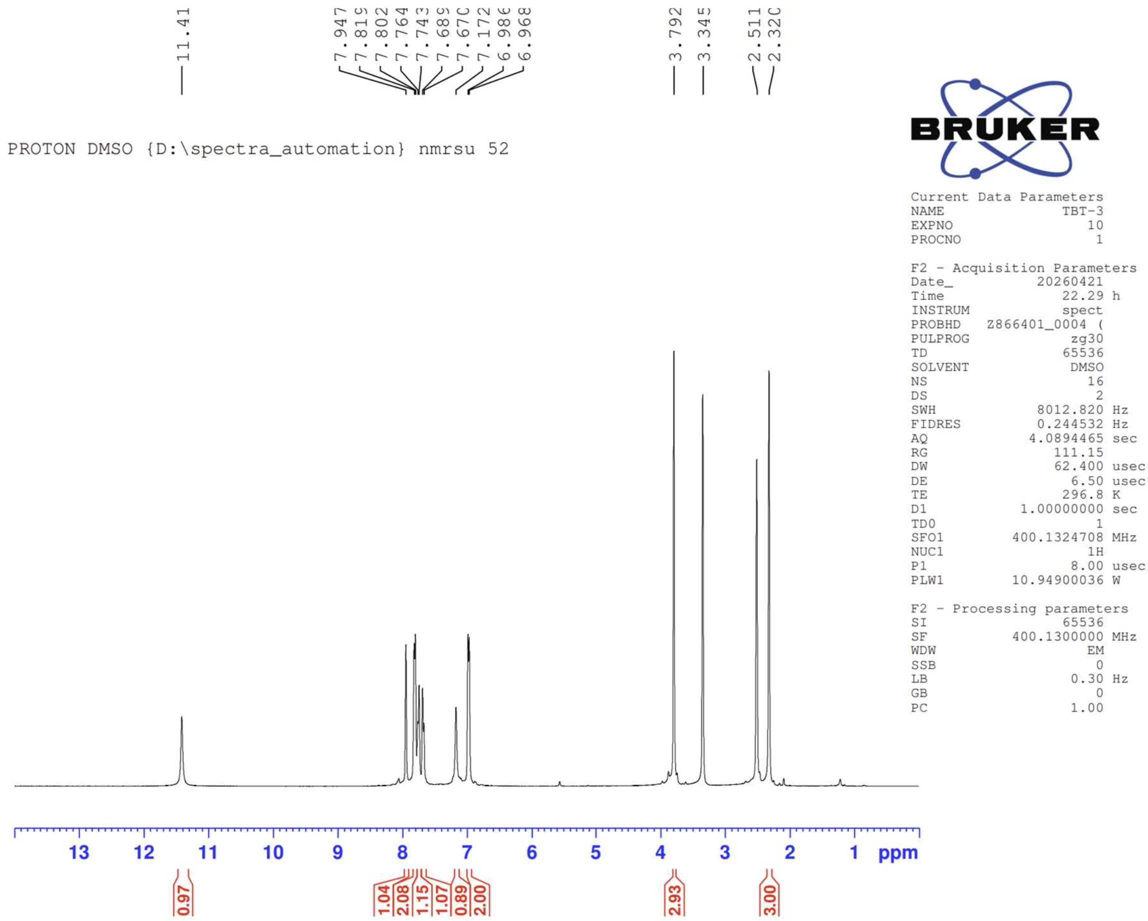

Figure S11. ^{1}H-NMR spectrum of compound **18**

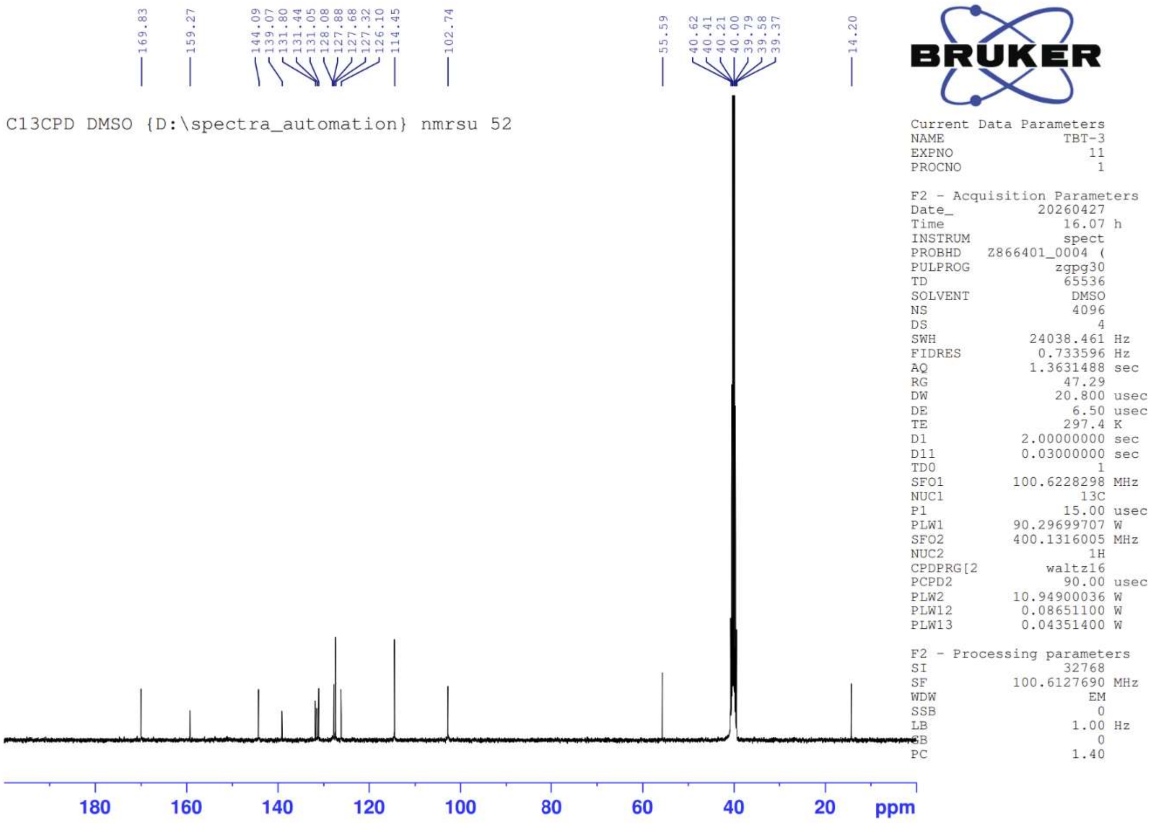

Figure S12. ^{13}C-NMR spectrum of compound **18**

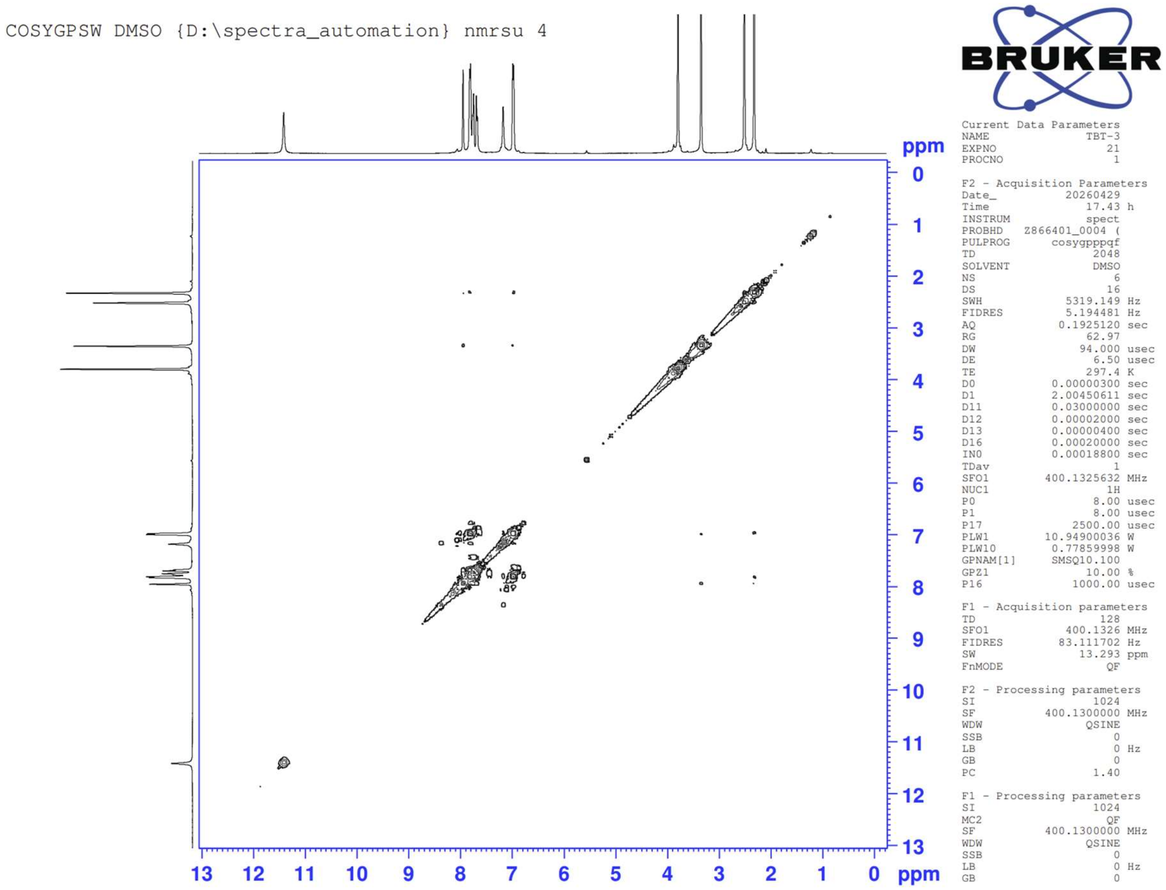

Figure S13. COSY spectrum of compound **18**

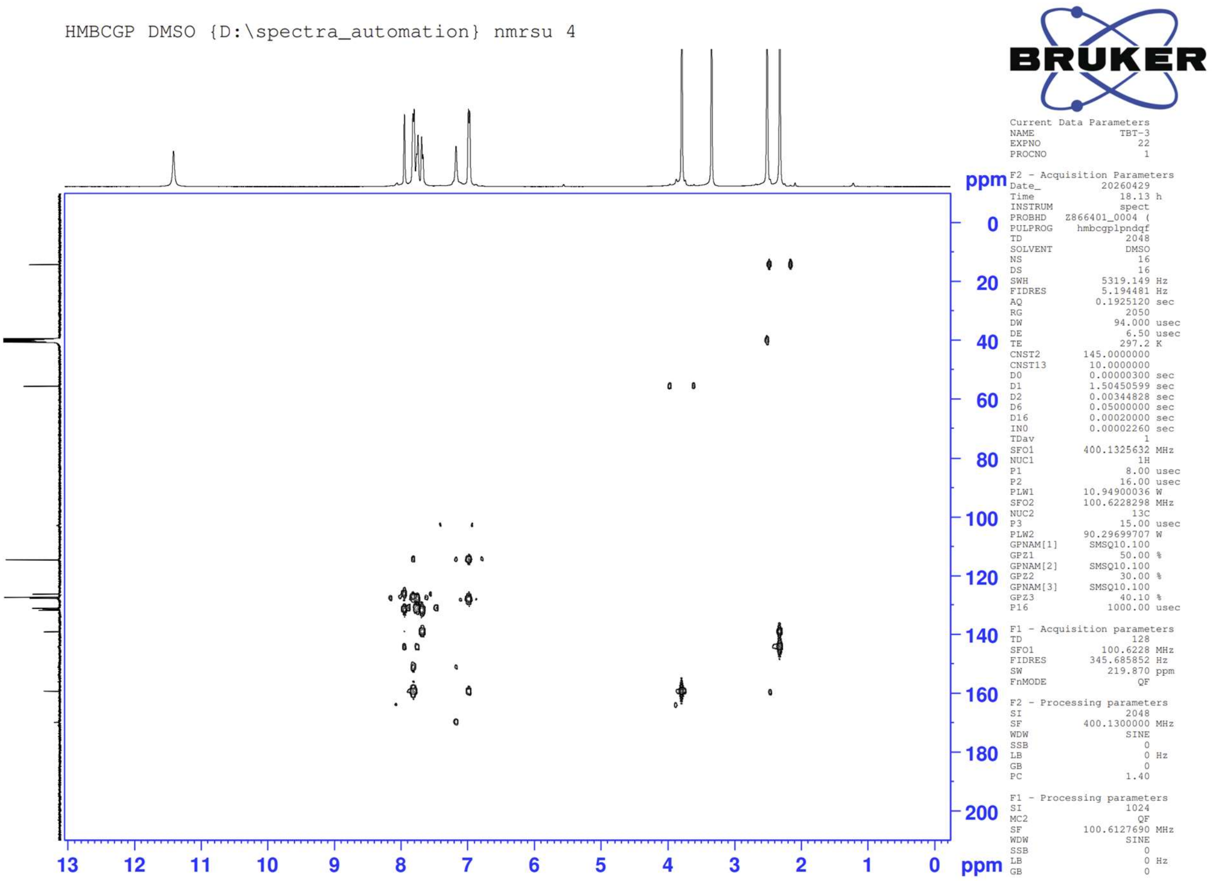

Figure S14. HMBC spectrum of compound **18**

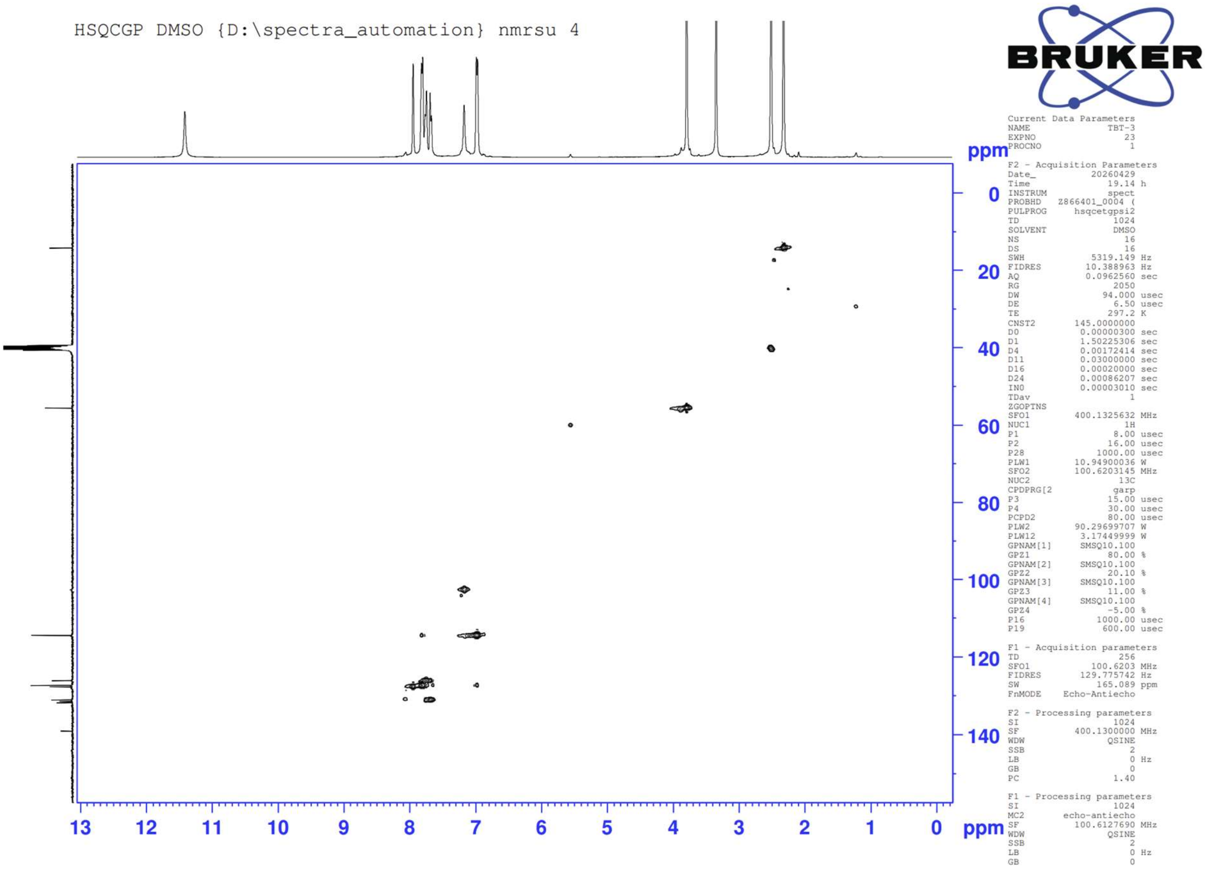

Figure S15. HSQC spectrum of compound **18**

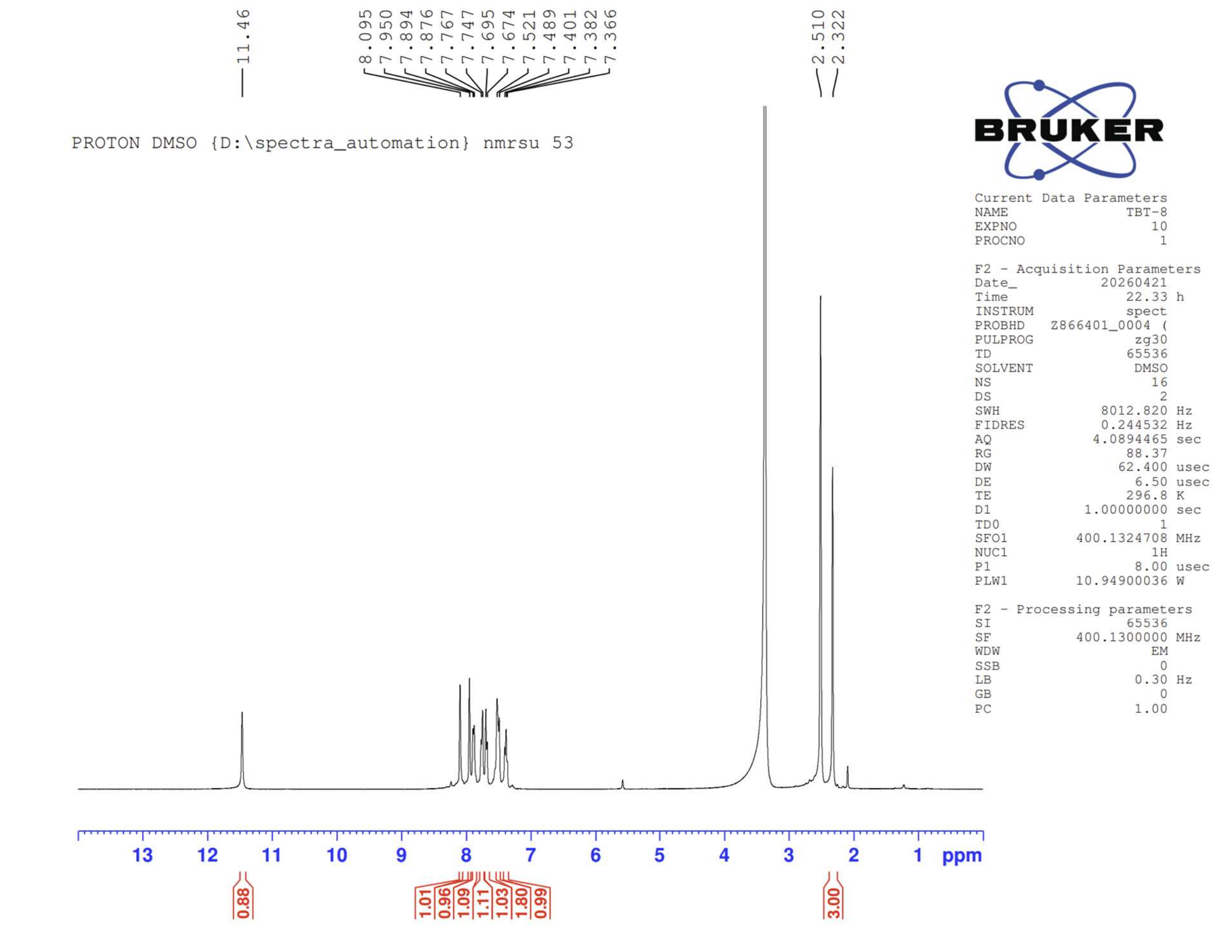

Figure S16. ^{1}H-NMR spectrum of compound **19**

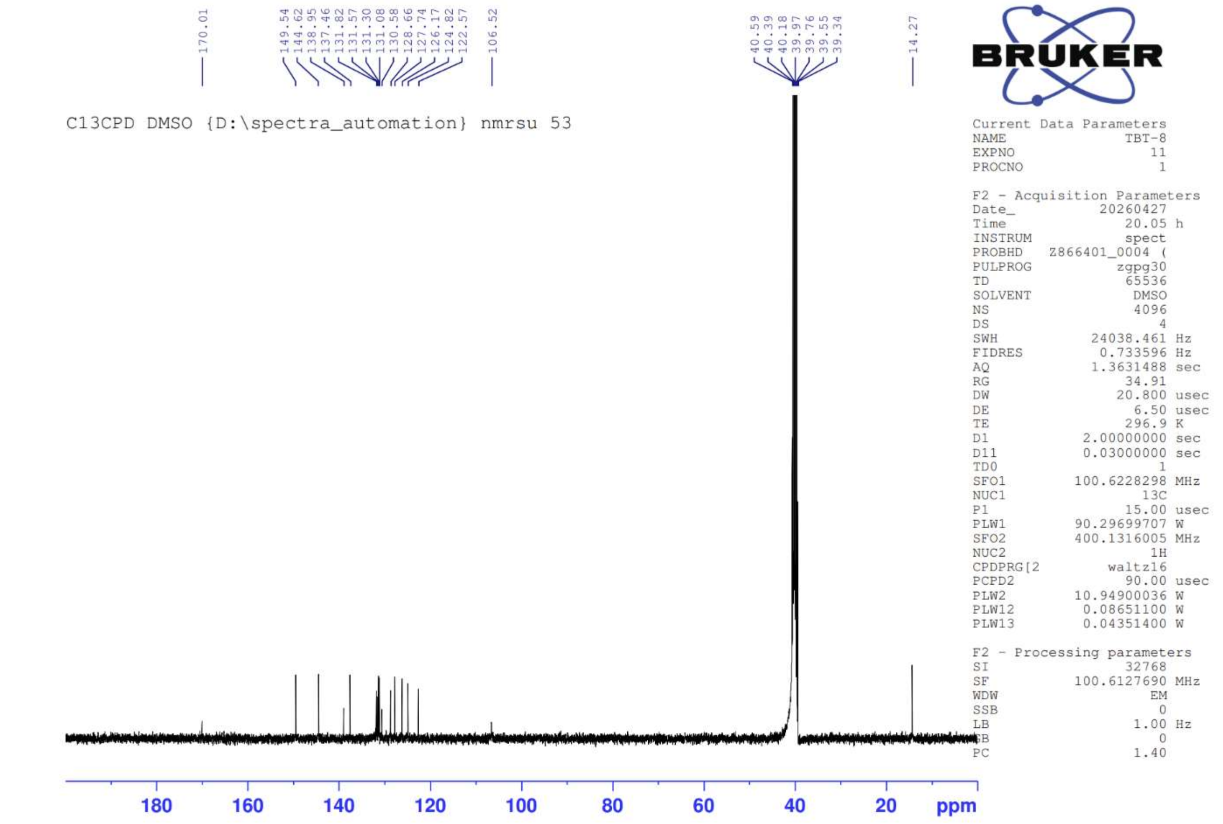

Figure S17. ^{13}C-NMR spectrum of compound **19**

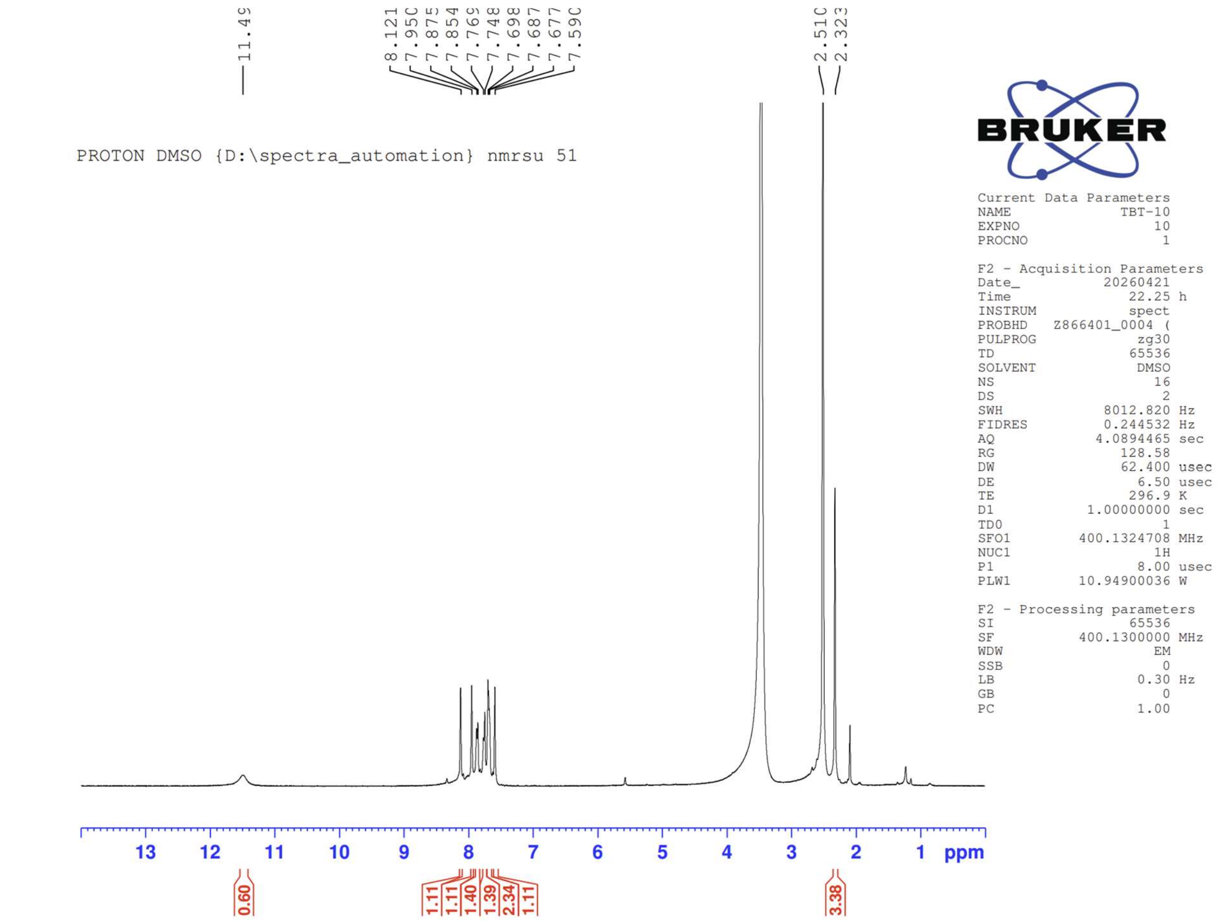

Figure S18. ^{1}H-NMR spectrum of compound **20**

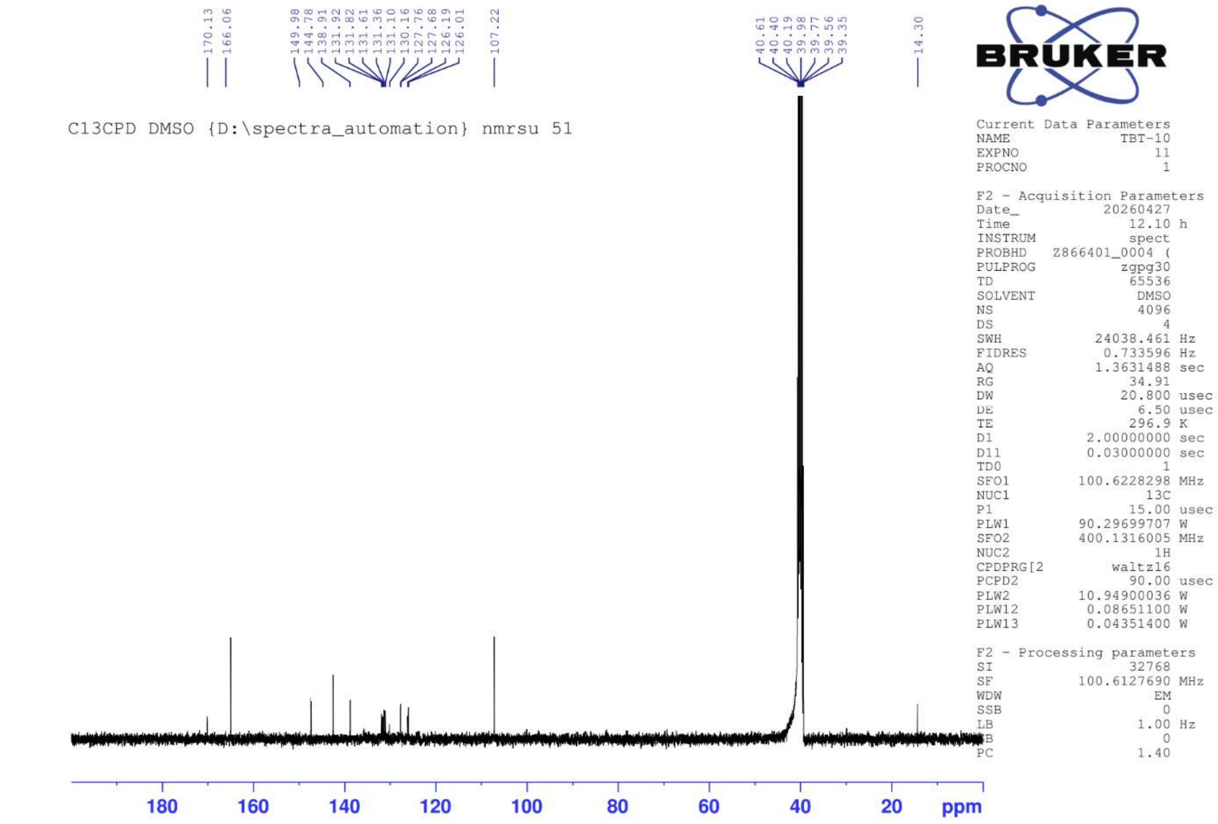

Figure S19. ^{13}C-NMR spectrum of compound **20**

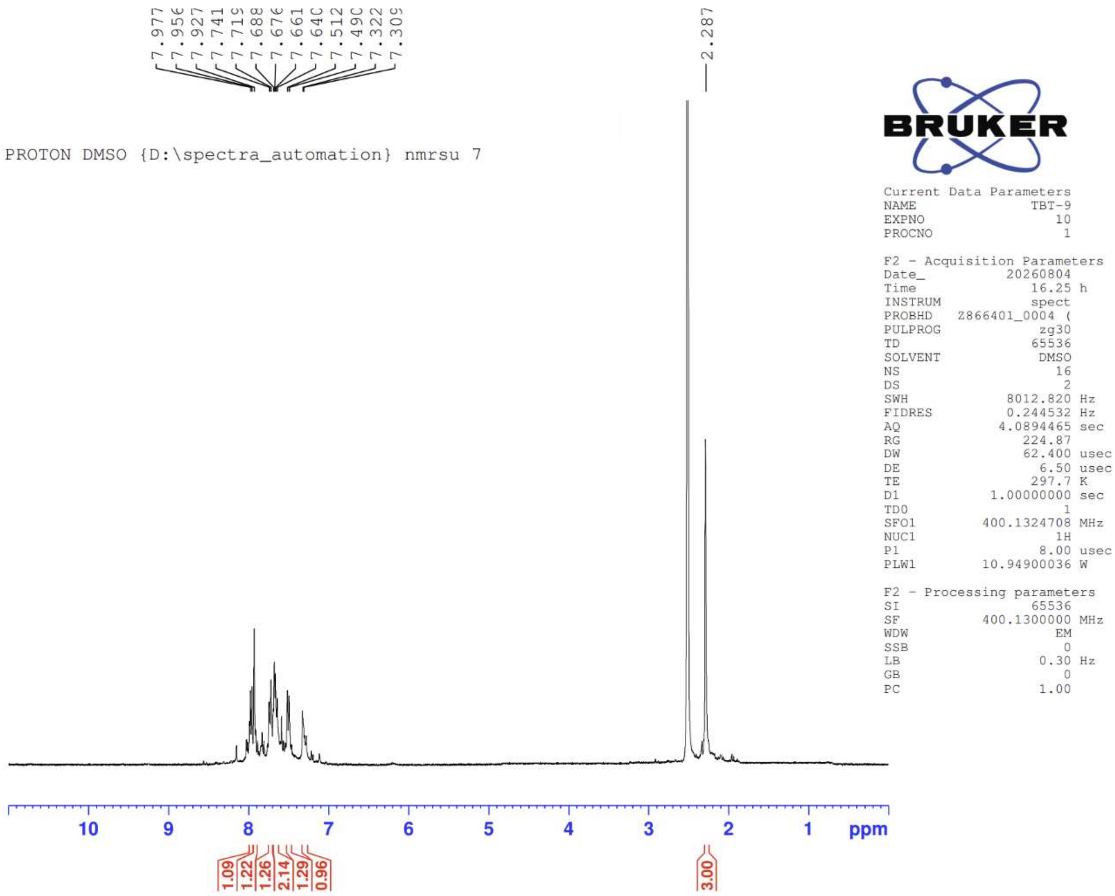

Figure S20. ^{1}H-NMR spectrum of compound **21**

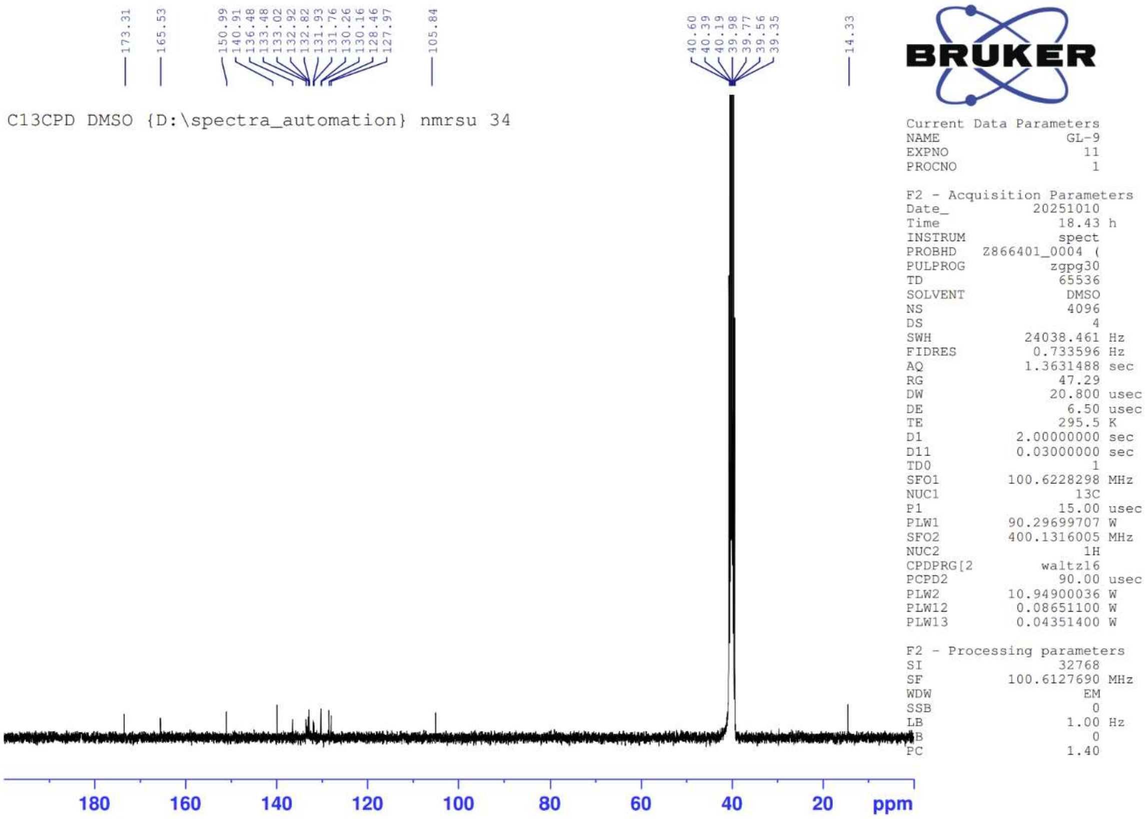

Figure S21. ^{13}C-NMR spectrum of compound **21**

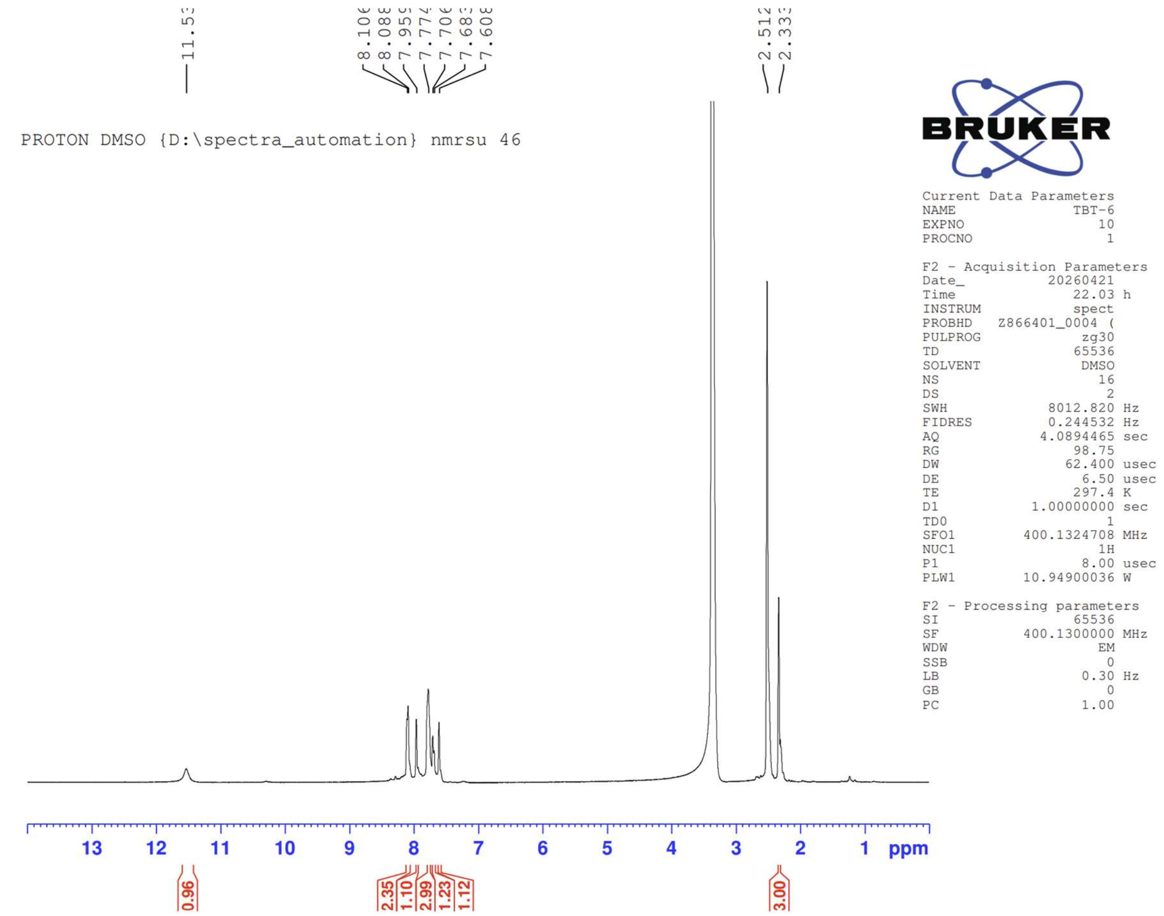

Figure S22. ^{1}H-NMR spectrum of compound **22**

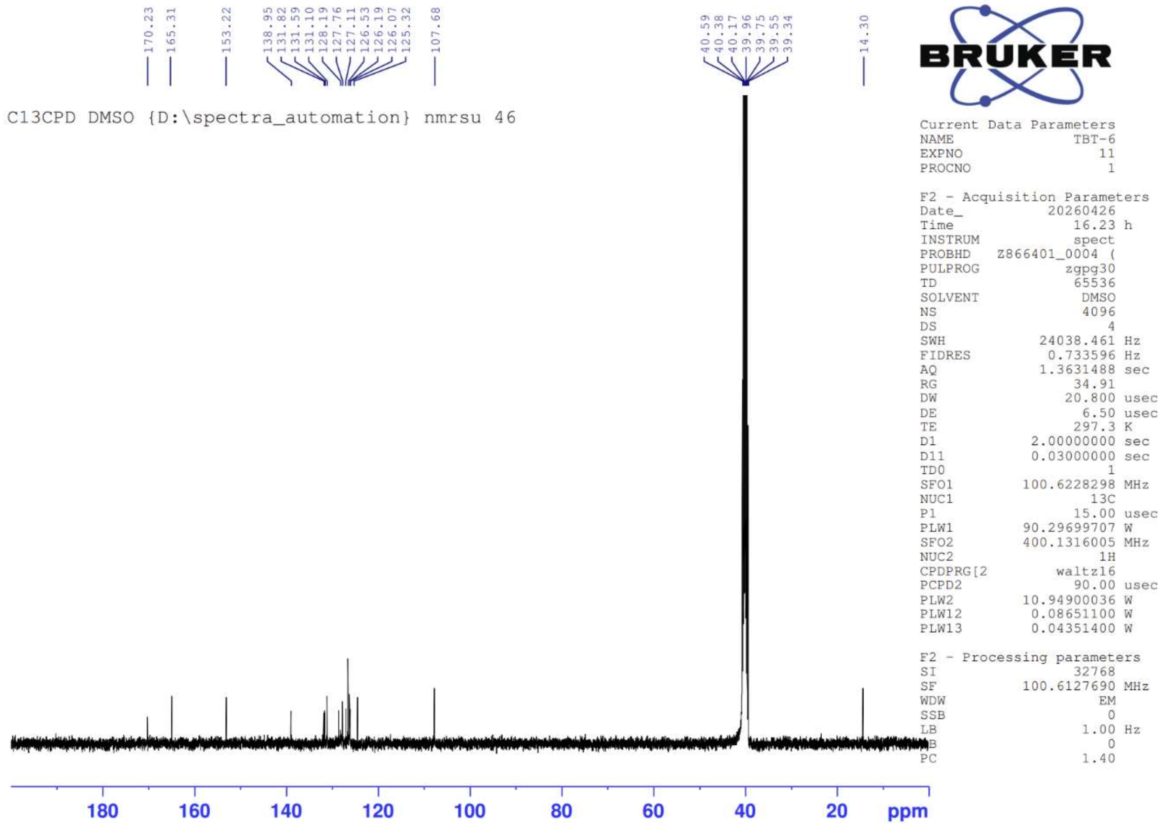

Figure S23. ^{13}C-NMR spectrum of compound **22**

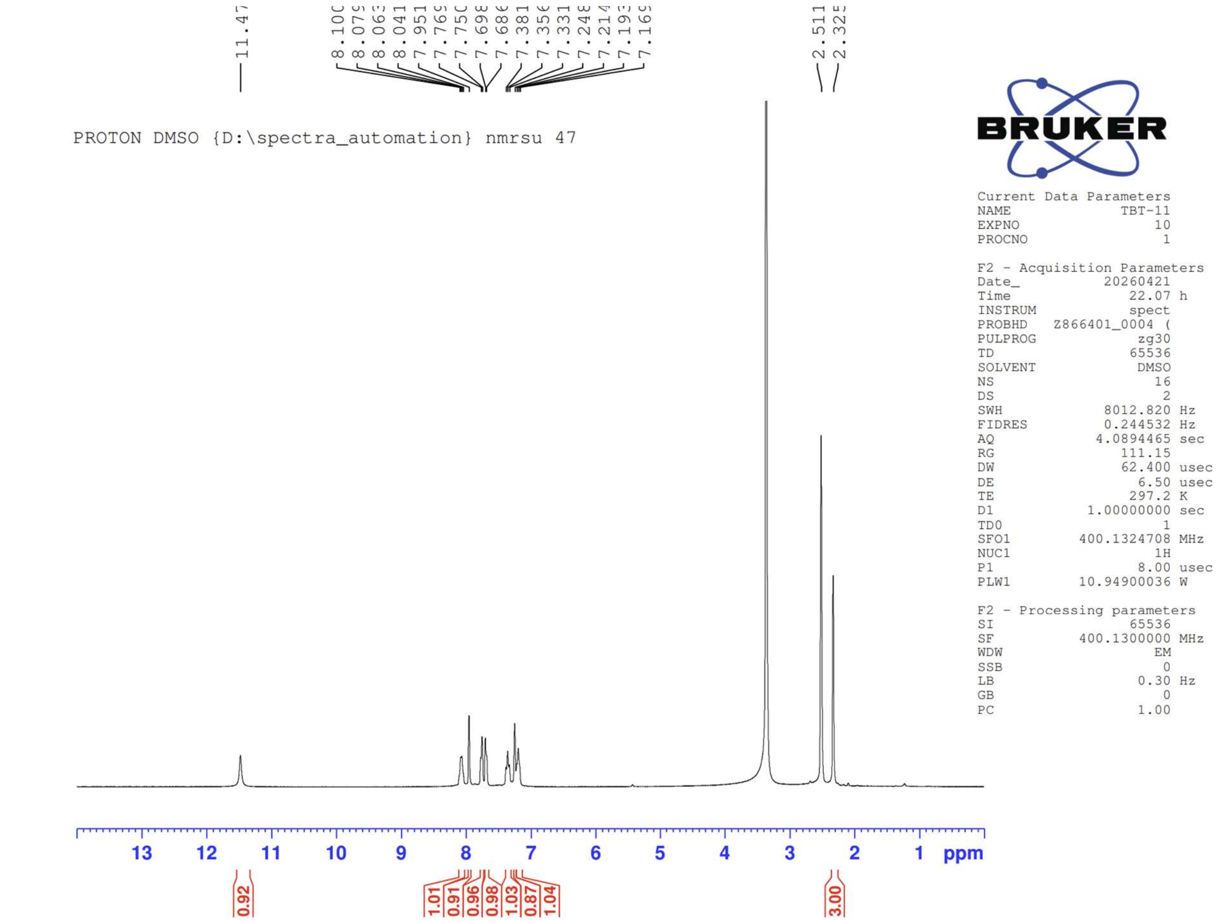

Figure S24. ^{1}H-NMR spectrum of compound **23**

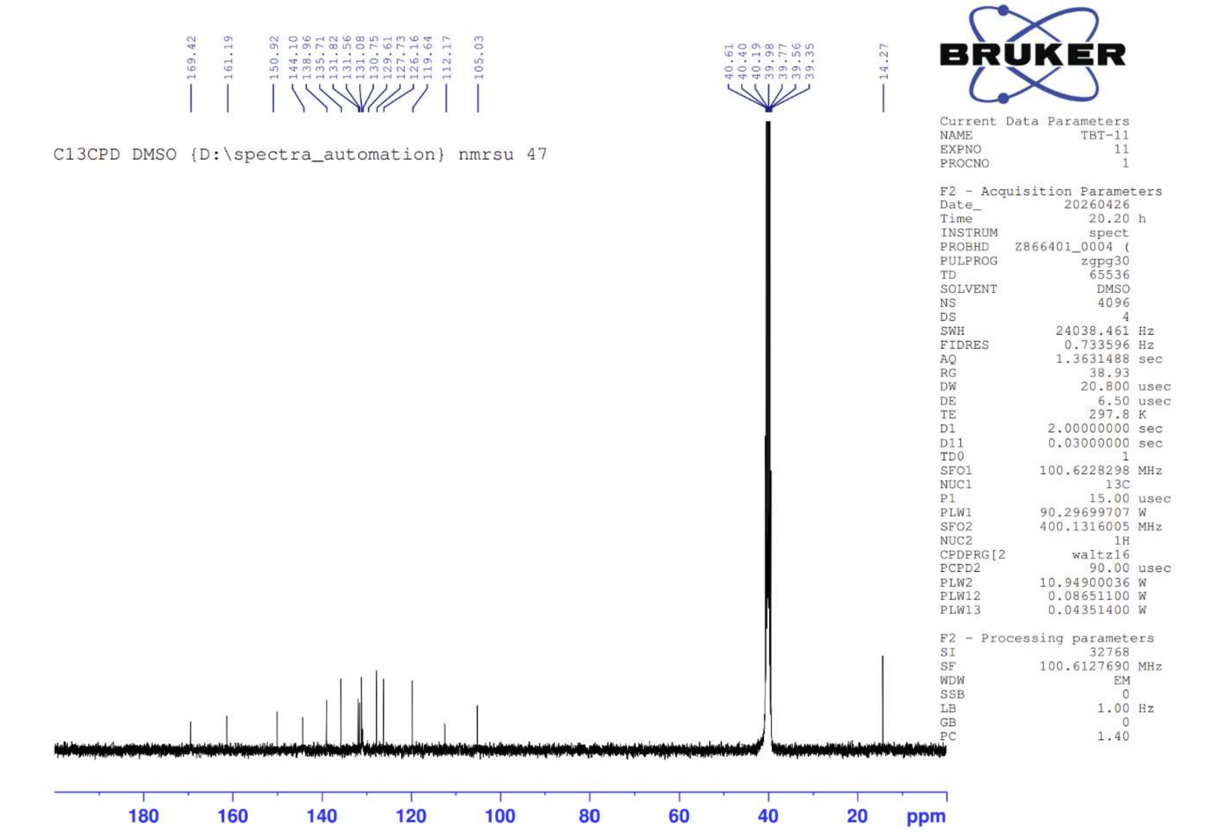

Figure S25. ^{13}C-NMR spectrum of compound **23**
